# Complete genome sequence of DSM 30083^T^, the type strain (U5/41^T^) of *Escherichia coli*, and a proposal for delineating subspecies in microbial taxonomy

**DOI:** 10.1186/1944-3277-9-2

**Published:** 2014-12-08

**Authors:** Jan P Meier-Kolthoff, Richard L Hahnke, Jörn Petersen, Carmen Scheuner, Victoria Michael, Anne Fiebig, Christine Rohde, Manfred Rohde, Berthold Fartmann, Lynne A Goodwin, Olga Chertkov, TBK Reddy, Amrita Pati, Natalia N Ivanova, Victor Markowitz, Nikos C Kyrpides, Tanja Woyke, Markus Göker, Hans-Peter Klenk

**Affiliations:** Leibniz Institute DSMZ – German Collection of Microorganisms and Cell Cultures, Inhoffenstraße 7B, 38124 Braunschweig, Germany; Helmholtz Centre for Infection Research, Inhoffenstraße 7, 38124 Braunschweig, Germany; LGC Genomics GmbH, Ostendstraße 25, 12459 Berlin, Germany; DOE Joint Genome Institute, Walnut Creek, Ca USA; Department of Biological Sciences, King Abdulaziz University, Jeddah, Saudi Arabia

**Keywords:** Phylogenomics, Phylotypes, GBDP, OPM, Phenotype, Clustering, Supermatrix, DNA:DNA hybridization, G+C content

## Abstract

Although *Escherichia coli* is the most widely studied bacterial model organism and often considered to be the model bacterium *per se*, its type strain was until now forgotten from microbial genomics. As a part of the ***G****enomic****E****ncyclopedia of****B****acteria and****A****rchaea* project, we here describe the features of *E. coli* DSM 30083^T^ together with its genome sequence and annotation as well as novel aspects of its phenotype. The 5,038,133 bp containing genome sequence includes 4,762 protein-coding genes and 175 RNA genes as well as a single plasmid. Affiliation of a set of 250 genome-sequenced *E. coli* strains, *Shigella* and outgroup strains to the type strain of *E. coli* was investigated using digital DNA:DNA-hybridization (dDDH) similarities and differences in genomic G+C content. As in the majority of previous studies, results show *Shigella* spp. embedded within *E. coli* and in most cases forming a single subgroup of it. Phylogenomic trees also recover the proposed *E. coli* phylotypes as monophyla with minor exceptions and place DSM 30083^T^ in phylotype B2 with *E. coli* S88 as its closest neighbor. The widely used lab strain K-12 is not only genomically but also physiologically strongly different from the type strain. The phylotypes do not express a uniform level of character divergence as measured using dDDH, however, thus an alternative arrangement is proposed and discussed in the context of bacterial subspecies. Analyses of the genome sequences of a large number of *E. coli* strains and of strains from > 100 other bacterial genera indicate a value of 79-80% dDDH as the most promising threshold for delineating subspecies, which in turn suggests the presence of five subspecies within *E. coli*.

**Electronic supplementary material:**

The online version of this article (doi:10.1186/1944-3277-9-2) contains supplementary material, which is available to authorized users.

## Introduction

Despite more than 35,000 completed and ongoing bacterial genome-sequencing projects (including over 2,500 genomes from strains of the genus Escherichia) [1] and the fundamental importance of type strains for microbial taxonomy and nomenclature [2], the type strain of *Escherichia coli*, U5/41^T^, the most widely studied bacterial model organism and model bacterium *per se*, was until now neglected in microbial genomics; although strain K-12 substrain MG1665 was in 1997 the subject of one of the first ever published complete genome sequences [3]. By sequencing the genome of DSM 30083^T^, DSMZ’s culture of U5/41^T^, in the context of the Genomic Encyclopedia of *Bacteria* and *Archaea* [4], we filled this gap enabling not only the use of this strain as a taxonomic reference in genome sequence-based studies, but also providing access to novel data of an exciting organism whose phenotypic features differ in many ways from those of the often used *E. coli* lab strain K-12.

The first report on strains of the genus Escherichia (at that time termed “*Bacterium coli commune”*) were published in 1886 by Theodor Escherich [5] in the context of his professorial dissertation at University of Munich. Later in 1919, Castellani and Chalmers proposed the name *Escherichia coli* (*E.sche.ri’chi.a*, M.L. fem.n., *Escherichia,* in honor of Theodor Escherich; *co’li,* Gr.n. colon large intestine, colon, M.L. gen.n. *coli* of the colon) as the name for the type species of the genus Escherichia, which was accepted by the *Judical Commission of the ICSB* in 1958 [6] and included in the *Approved Lists of Bacterial Names* in 1980 [7].

**Figure 1:**
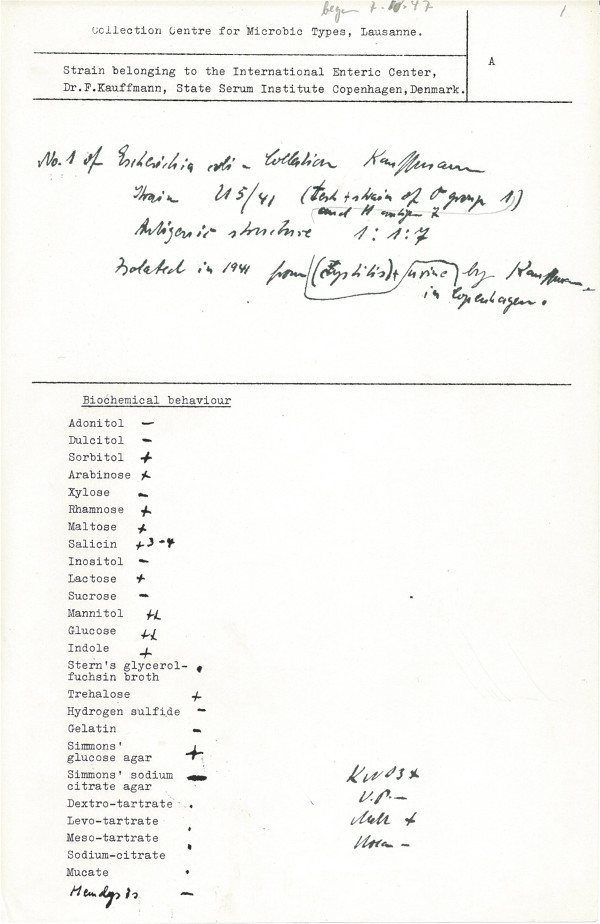
**Scan of the original record card issued for the deposit of U5/41** ^**T**^ **.**

Despite its enormous importance for microbiology and mostly due to a lack of type culture collections until the early 1920s, the original type cultures of *E. coli* got lost (just like those of the early isolates of other bacterial species). Strain U5/41^T^ (= DSM 30083^T^ = ATCC 11775^T^ = WDCM 00090^T^) was isolated by Fritz Kauffmann at the State Serum Institute Copenhagen, Denmark in 1941 [8], from the urine of a patient with cystitis, and was accepted as neotype of *E. coli* in 1963 [9]. Figure 1 shows the original record card issued by the Danish State Serum Institute in Copenhagen for the deposit of U5/41^T^. Since then, *E. coli* DSM 30083^T^ was a reference strain for many tests and applications, such as serotyping with the method of Ã˜rskov and Ã˜rskov [10], antimicrobial assays [11], ribotyping and multi-locus sequence typing [12], and the PCR amplification of the *β*-d-glucuronidase gene fragment (*uidA*) as tracer for fecal pollution in all kinds of waters [13]. As a model organism for genetics, biochemistry, metabolic reconstruction and pathway inference, genomics and metabolics of *E. coli* are well-studied topics, starting with the 1997 publication of the K-12 genome [3]. The reader is referred to studies of *E. coli* such as metabolic engineering for the production of chemicals and biofuels [14, 15], recombinant protein expression [16], the process of binary fission [17], DNA replication and segregation [18], small RNA regulators [19], genetics of the capsular machinery gene cluster [20], as well as comparative genomics [21] and the current status and the progress in clinically relevant *E. coli* strains [22, 23].

In this study we analyzed the genome sequence of *E. coli* DSM 30083^T^. We present a description of the genome sequencing and annotation and a summary classification together with a set of features for strain DSM 30083^T^, including novel aspects of its phenotype. Since only the availability of the type-strain genome allows for the application of state-of-the-art genome-based taxonomic methods, species affiliation of all strains with respect to the type strain was determined *via* digital DNA:DNA-hybridization (dDDH) similarities as computed by the Genome-to-Genome Distance Calculator [24], version 2 [25], and by evaluating the differences in genomic G+C content [26]. Phylogenomic analyses [24, 25] elucidate the evolutionary relationships between 250 *E. coli* strains, *Shigella* spp. and outgroup strains as well as the grouping within *E. coli*. The availability of the type-strain genome allows not only for assessing whether published genome sequences are actually from strains of *E. coli* but also for a potential division of *E. coli* into subspecies.

## Organism features

### Classification and features

#### 16S rRNA gene analysis

The sequences of the seven 16S rRNA gene copies in the genome of DSM 30083^T^ differ from each other by up to eleven nucleotides, and differ by up to ten nucleotides from the previously published 16S rRNA gene sequence (X80725), which contains three ambiguous base calls. The phylogenetic neighborhood of *E. coli* in a 16S rRNA gene-based tree inferred as previously described [27] is shown in Additional file 1.

The single genomic 16S rRNA gene sequence of *E. coli* DSM 30083^T^ was compared with the Greengenes database for determining the weighted relative frequencies of taxa and (truncated) keywords as previously described [27]. The most frequently occurring genera were *Escherichia* (87.0%) and *Shigella* (13.0%) (131 hits in total). Regarding the 109 hits to sequences from representatives of the species, the average identity within HSPs was 99.8%, whereas the average coverage by HSPs was 100.0%. Regarding the five hits to sequences from other representatives of the genus, the average identity within HSPs was also 99.8%, whereas the average coverage by HSPs was 100.0%. Among all other species, the one yielding the highest score was *Shigella flexneri* (HQ407229), which corresponded to an identity of 99.9% and an HSP coverage of 100.0%. (Note that the Greengenes database uses the INSDC (=EMBL/NCBI/DDBJ) annotation, which is not an authoritative source for nomenclature or classification.) The highest-scoring environmental sequence was EF603461 (Greengenes short name ‘Salmonella typhimurium Exploits Inflammation Compete Intestinal Microbiota mouse cecum clone 16saw29-1c11.q1k’), which showed an identity of 99.9% and an HSP coverage of 100.0%. The most frequently occurring keywords within the labels of all environmental samples which yielded hits were ‘intestin’ (9.9%), ‘mous’ (6.1%), ‘inflamm’ (5.8%), ‘microbiota’ (5.7%) and ‘cecum, compet, exploit, salmonella, typhimurium’ (5.6%) (119 hits in total). The most frequently occurring keywords within the labels of those environmental samples which yielded hits of a higher score than the highest scoring species were ‘microbiota’ (12.5%), ‘cecum, compet, exploit, inflamm, intestin, mous, salmonella, typhimurium’ (10.0%) and ‘gut, lusitanicu, thorect’ (2.5%) (5 hits in total). These keywords fit well to the known ecology of *E. coli*.

#### Morphology and physiology

**Figure 2:**
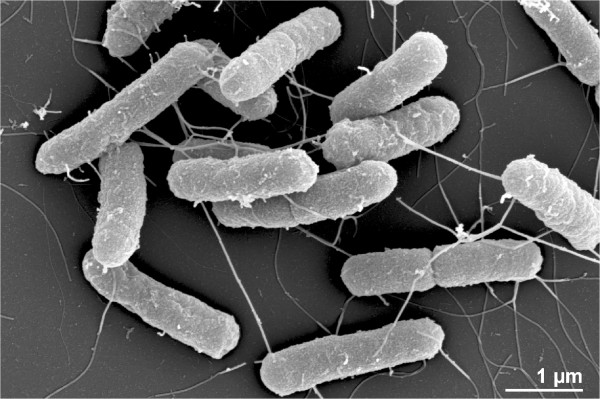
**Scanning-electron micrograph of strain** ***E. coli*** **DSM 30083** ^**T**^ **.**

As described for the genus Escherichia, cells are Gram-negative, medium to long rods (Figure 2 and Table 1), motile by the means of peritrichous flagella, non-pigmented, chemo-organotrophic, oxidase-negative, facultative anaerobes. They produce acid and gas while fermenting d-glucose, lactose or other carbohydrates [28]. *E. coli* strains are able to grow at temperatures between 10°C and 45°C, with an optimum between 37°C and 42°C, and at pH 5.5-8.0 [28, 29]. Koser [30] showed that “*Bacterium coli communis”* utilizes propionic acid, n-butyric acid, succinic acid, malic acid, lactic acid and mucic acid as sole carbon sources, but neither citric acid, salts of citric acid, n- or iso-valeric acid, n-caprionic acid, tartaric acid, oxalic acid, benzoic acid, salicylic acid nor o-phthalic acid. Based on the description by Kauffmann (Figure 1), strain DSM 30083^T^ grows on d-trehalose, d-sorbitol, d-mannitol, l-rhamnose, d-glucose, d-maltose, α-d-lactose, d-arabinose, but does not grow on dulcitol, d-xylose, sucrose, adonitol, citric acid, inositol and gelatin and growth varies on d-salicin. Strain DSM 30083^T^ belongs to *E. coli* “var. *communis”* (representatives were mostly isolated from feces), because the strain does not ferment sucrose or salicin [31]. Strain DSM 30083^T^ is able to ferment lactose (Figure 1), which is a characteristic criterion for the differentiation against representatives of *Shigella* and *Salmonella* [28, 29]. Comparable to most strains of *E. coli*, strain DSM 30083^T^ is positive for indole production, nitrate reduction, and urease but hydrogen-sulfide negative (Figure 1) [28]. Additionally, Huys et al. [32] described strain ATCC 11775^T^ as being positive for d-raffinose and acetate utilization, positive for lysin-decarboxylase and ornithine-decarboxylase activity, and negative for growth on d-arabitol, d-cellobiose and in the presence of KCN. Furthermore, *E. coli* utilizes mucic acid, does not produce acetoin (Voges–Proskauer negative), and does not utilize malonate [29].

**Table 1:** **Classification and general features of** ***E. coli*** **DSM 30083**^**T**^ **in accordance with the MIGS recommendations** [33] **published by the Genome Standards Consortium** [34]

MIGS ID	Property	Term	Evidence code
	Current classification	Domain *Bacteria*	TAS [35]
		Phylum *Proteobacteria*	TAS [36]
		Class *Gammaproteobacteria*	TAS [37, 38]
		Order '*Enterobacteriales*'	TAS [37, 38]
		Family *Enterobacteriaceae*	TAS [39]
		Genus *Escherichia*	TAS [5, 9]
		Species *Escherichia coli*	TAS [5, 9]
		Strain U5/41^T^	TAS [5, 9, 40]
		Serovar: O1:K1(L1):H7	IDA, TAS [10]
	Gram stain	Negative	IDA, TAS [28]
	Cell shape	Rod	TAS [28]
	Motility	Motile	TAS [28]
	Sporulation	Non-sporeforming	IDA, TAS [28]
	Temperature range	Mesophile	NAS
MIGS-6.1	Optimum temperature	37°C	IDA, TAS [41]
MIGS-6.3	Salinity range	Not reported	
MIGS-22	Oxygen requirement	Aerobe and facultative anaerobe	TAS [28, 29]
	Carbon source	Carbohdrates, salicin, sorbitol, mannitol, indole, peptides	IDA, TAS [41], (Figure 1)
	Energy metabolism	Chemo-organotrophic	TAS, IDA [29]
MIGS-15	Biotic relationship	Human specimen	NAS
MIGS-14	Pathogenicity	Human and animal	NAS
	Biosafety level	2	TAS [42]
MIGS-23	Isolation	Urine	TAS (Figure 1)
MIGS-23	Cultivation	Nutrient agar (DSMZ medium 1)	IDA, TAS [41]
MIGS-4	Geographic location	Copenhagen, Denmark	TAS (Figure 1)
	Collected by	F. Kauffmann	TAS (Figure 1)
MIGS-5	Sample collection time	1941	TAS (Figure 1)
MIGS-4.1 MIGS-4.2	Latitude – Longitude	55° 40′ 34″ N, 12° 34′ 6″ E	TAS (Figure 1)

Evidence codes - TAS: Traceable Author Statement (i.e., a direct report exists in the literature); NAS: Non-traceable Author Statement (i.e., not directly observed for the living, isolated sample, but based on a generally accepted property for the species, or anecdotal evidence); IDA: Inferred from direct assay. Evidence codes are from of the Gene Ontology project [43].

We used phenotyping with the OmniLog instrument [Biolog Inc., Hayward, CA] to elucidate whether or not strain DSM 30083^T^ might be able to utilize further substrates. A comparison of *E. coli* DSM 30083^T^ and *E. coli* DSM 18039 (a K-12 MG1655 derivative with almost K-12 wild-type features) with Generation-III microplates run in an OmniLog phenotyping instrument was conducted by Vaas *et al.* [44]. These data also serve as exemplars for the substrate-information and feature-selection facilities in the tutorial of the opm package [45] for analyzing phenotype microarray data in the R statistical environment [46]. As shown in that tutorial, among the substrates contained in Generation-II plates, carbohydrates make the main difference between the two strains, with DSM 30083^T^ mostly reacting more strongly than DSM 18039.

The utilization of carbon compounds by *E. coli* DSM 30083^T^ grown at 37°C in LB medium (DSMZ medium no. 381) [41] was also determined for this study using PM-01 and PM-02 microplates [Biolog Inc., Hayward, CA]. These plates were inoculated at 37°C with dye A and a cell suspension at a cell density of 85% turbidity. The exported measurement data were further analyzed with opm using its functionality for statistically estimating parameters from the respiration curves such as the maximum height, and automatically translating these values into negative, ambiguous, and positive reactions. The reactions were recorded in two individual biological replicates, and results that differed between the two replicates were regarded as ambiguous.

On PM-01 microplates, DSM 30083^T^ was positive for l-arabinose, *N*-acetyl-d-glucosamine, d-saccharic acid, succinic acid, d-galactose, l-aspartic acid, l-proline, d-alanine, d-trehalose, d-mannose, d-serine, d-sorbitol, glycerol, l-fucose, d-glucuronic acid, d-gluconic acid, d,l-α-glycerol-phosphate, l-lactic acid, d-mannitol, l-glutamic acid, d-glucose-6-phosphate, d-galactonic acid-γ-lactone, d,l-malic acid, d-ribose, tween 20, l-rhamnose, d-fructose, acetic acid, d-glucose, d-maltose, d-melibiose, thymidine, l-asparagine, d-glucosaminic acid, tween 40, α-keto-glutaric acid, α-methyl-d-galactoside, α-d-lactose, lactulose, uridine, l-glutamine, α-d-glucose-1-phosphate, d-fructose-6-phosphate, β-methyl-d-glucoside, maltotriose, 2′-deoxy-adenosine, adenosine, gly-asp, fumaric acid, bromo-succinic acid, propionic acid, glycolic acid, glyoxylic acid, inosine, gly-glu, l-serine, l-threonine, l-alanine, ala-gly, *N*-acetyl-β-d-mannosamine, mono-methyl succinate, methyl pyruvate, d-malic acid, l-malic acid, gly-pro, l-lyxose, glucuronamide, pyruvic acid, l-galactonic acid-γ-lactone and d-galacturonic acid.

The strain was negative for the negative control, dulcitol, d-xylose, d-aspartic acid, α-keto-butyric acid, sucrose, *m*-tartaric acid, tween 80, α-hydroxy-glutaric acid-γ-lactone, α-hydroxy-butyric acid, adonitol, citric acid, myo-inositol, d-threonine, mucic acid, d-cellobiose, tricarballylic acid, acetoacetic acid, *p*-hydroxy-phenylacetic acid, *m*-hydroxy-phenylacetic acid, tyramine, d-psicose, β-phenylethylamine and ethanolamine.

Ambiguous results were obtained with sodium formate and 1,2-propanediol.

On PM-02 microplates, DSM 30083^T^ was positive for dextrin, *N*-acetyl-d-galactosamine, *N*-acetyl-neuraminic acid, β-d-allose, d-arabinose, 3-*O*-β-d-galactopyranosyl-d-arabinose, d-lactitol, β-methyl-d-galactoside, β-methyl-d-glucuronic acid, d-raffinose, l-sorbose, d-tagatose, d-glucosamine, β-hydroxy-butyric acid, d-lactic acid methyl ester, melibionic acid, l-alaninamide and dihydroxy-acetone.

The strain was negative for the negative control, chondroitin sulfate C, α-cyclodextrin, β-cyclodextrin, γ-cyclodextrin, gelatin, glycogen, inulin, laminarin, mannan, pectin, amygdalin, d-arabitol, l-arabitol, arbutin, 2-deoxy-d-ribose, *m*-erythritol, d-fucose, β-gentiobiose, l-glucose, d-melezitose, maltitol, α-methyl-d-glucoside, 3-*O*-methyl-d-glucose, α-methyl-d-mannoside, β-methyl-d-xylopyranoside, palatinose, d-salicin, sedoheptulosan, stachyose, turanose, xylitol, *N*-acetyl-d-glucosaminitol, γ-amino-n-butyric acid, Î´-amino-valeric acid, butyric acid, capric acid, caproic acid, citraconic acid, d-citramalic acid, 2-hydroxy-benzoic acid, 4-hydroxy-benzoic acid, γ-hydroxy-butyric acid, α-keto-valeric acid, itaconic acid, 5-keto-d-gluconic acid, malonic acid, oxalic acid, oxalomalic acid, quinic acid, d-ribono-1,4-lactone, sebacic acid, sorbic acid, succinamic acid, d-tartaric acid, l-tartaric acid, acetamide, *N*-acetyl-l-glutamic acid, l-arginine, glycine, l-histidine, l-homoserine, l-hydroxyproline, l-isoleucine, l-leucine, l-lysine, l-methionine, l-ornithine, l-phenylalanine, l-pyroglutamic acid, l-valine, d,l-carnitine, butylamine (sec), d,l-octopamine, putrescine, 2,3-butanediol, 2,3-butanedione and 3-hydroxy-2-butanone. Ambiguous results were not observed on PM-02 microplates.

Results of the OmniLog phenotyping in PM-01 and PM-02 microplates (see Additional file 1 for further information) were in full agreement with growth experiments as described in the aforementioned literature with the sole exception of mucic acid [29], which was not metabolized by strain DSM 30083^T^ in OmniLog phenotyping, at least not within the applied running time. In brief, strain DSM 30083^T^ grows on succinic acid, d-sorbitol, l-lactic acid, d-mannitol, l-rhamnose, acetic acid, d-glucose, d-maltose, α-d-lactose, propionic acid, d-trehalose, d-malic acid, l-malic acid, d-arabinose, and d-raffinose, but does not grow on dulcitol, d-xylose, sucrose, *m*-tartaric acid, adonitol, citric acid, myo-inositol, d-cellobiose, gelatin, d-arabitol, d-salicin, butyric acid, malonic acid, oxalic acid, d-tartaric acid, and l-tartaric acid. Strain DSM 30083^T^ grows on d-galacturonic acid, d-glucuronic acid, α-keto-glutaric acid and glutamic acid, which suggests a catabolism of d-glucuronic acid and d-galacturonic acid to α-keto-glutaric acid and further to glutamic acid *via* the mucic-acid pathway [47, 48].

We tested growth on further substrates by incubating strain DSM 30083^T^ either on DSMZ medium 382 (M9) without glucose [41], supplemented with 20 mM substrate at 37°C for 72 h, or with API 20E strips (bioMérieux, Nürtingen, Germany) at 37°C. On API 20E strips (see Additional file 1) strain DSM 30083^T^ was positive for β-galactosidase, l-lysine, l-ornithine, indole production, d-glucose, d-mannitol, d-sorbitol, l-rhamnose, d-melibiose, and l-arabinose, but negative for l-arginine, citrate, sulfide production, urease, l-tryptophane, acetoin production, gelatin, inositol, sucrose, amygdaline, and oxidase. In medium M9 strain DSM 30083^T^ showed growth on l-glutamic acid, tween 20, *N-* acetyl-d-galactosamine, l-sorbose, and d-melibiose, but not on 1,2-propanediol, dulcitol, d-xylose, *m-* tartaric acid, and α-keto-butyric acid. In experiments conducted at DSMZ, strain DSM 30083^T^ formed blue colonies on OXOID Brilliance ESBL Agar (P05302A, OXOID, UK) and utilized d-galactose and thus is both galactosidase- and glucuronidase-positive. Indicated by the positive result of pyruvic acid in the OmniLog phenotyping and the negative Voges–Proskauer test, strain DSM 30083^T^ is able to utilize pyruvate but does not produce acetoin, a carbon storage and an intermediate to avoid acidification during fermentation [49].

### Chemotaxonomy

To the best of our knowledge, data on the fatty acids or polar lipids of *E. coli* DSM 30083^T^ are not available in the literature.

For details on the extensively studied molecular structure and chemical composition of the *E. coli* cell wall the reader is referred to Scheutz and Strockbine [29] and the literature listed therein. In brief, *E. coli* has a single peptidoglycan layer within the periplasm, consisting of n-acetylglucosamine and n-acetylmuramic acid linked to the tetrapeptide l-alanine, d-glutamic acid, meso-diaminopimelic acid and d-alanine. The outer membrane is a lipopolysaccharide layer consisting of (i) lipid A, (ii) the core region of the phosphorylated nonrepeating oligosaccharides, and (iii) the O-antigen polymer [28, 29].

*E. coli*, *Shigella* ssp. and *Salmonella* ssp. strains display a huge variety of lipopolysaccharide layer heat-stable somatic (O), capsular (K; “Kapsel”, the German word for capsule), flagellar filament (H), and fimbriae (F) antigens, which serve since a long time as the basis for serotyping [29]. K antigens are further subdivided into the L, B, and A categories, based on their physical properties [29]. The serotype of *E. coli* DSM 30083^T^ is O1:K1(L1):H7.

Representatives of *E. coli*, as Gram-negative bacteria, are described to be intrinsically resistant to hydrophobic antibiotics (e.g. macrolites, novoviocins, rifamycins, actinomycin D, fusidic acid) and may have acquired further antibiotic resistances (e.g. aminoglycosides, β-lactam, chloramphenicol, sulfonamides, tetracyclines) [29]. We tested the antibiotic resistance of *E. coli* DSM 30083^T^ on Müller-Hinton agar at 30°C. Strain DSM 30083^T^ was resistant against the cell-envelope antibiotics bacitracin, oxacillin, penicillin G, teicoplanin and vancomycin as well as against the protein-synthesis inhibitors (50S subunit) clindamycin, lincomycin, linezolid, nystatin (antifungal) and quinupristin/dalfooristin. In contrast, strain DSM 30083^T^ was susceptible to the cell-envelope antibiotics ampicillin, azlocillin, aztreonam, cefalotin, cefazolin, cefotaxime, ceftriaxone, colistin, fosfomycin, imipenem, mezlocillin, piperacillin/tazobactam, ticarcillin and polymyxin B, the protein-synthesis inhibitors (30S subunit) amikacin, doxycyclin, gentamicin, kanamycin, neomycin and tetracyclin, the protein-synthesis inhibitors (50S subunit) chloramphenicol and erythromycin as well as against the nucleic-acid inhibitors moxifloxacin, nitrofurantoin, norflaxacin, oflaxacin and pipemidic acid.

As reported by F. Kauffmann (Figure 1) and tested at DSMZ on enterohaemolysin agar (PB5105A, OXOID, Wesel, Germany), strain DSM 30083^T^ is enterohaemolysin-negative and thus does not belong to enterohemorrhagic serotype (enterohaemorrhagic *E. coli*, EHEC). The T phages T_1_-T_7_ did not lyse strain DSM 30083^T^ cultivated on DSMZ medium 544 at 37°C.

## Genome sequencing and annotation

### Genome project history

The *E. coli* type strain genome was sequenced as part of the ***G****enomic****E****ncyclopedia of***B** acteria *and***A** rchaea (GEBA) project [4]. It was the only strain in the project that was chosen for genome sequencing due to its eminent prominence as a model organism and its value as a taxonomic reference strain and not selected according to the GEBA criteria for distinct phylogenetic location [4, 50]. Project information is found in the Genomes OnLine Database [1]. Draft sequencing, initial gap closure and annotation were performed by the DOE Joint Genome Institute (JGI) using state-of-the-art sequencing technology [51]. The Whole Genome Shotgun (WGS) sequence is deposited in Genbank and the Integrated Microbial Genomes database (IMG) [52]. A summary of the project information is shown in Table 2.

**Table 2:** **Genome sequencing project information**

MIGS ID	Property	Term
MIGS-31	Finishing quality	Level 3: Improved-High-Quality Draft
MIGS-28	Libraries used	454 Titanium paired-end, Solexa paired end
MIGS-29	Sequencing platforms	454-GS-FLX-Titanium, Illumina GAii
MIGS-31.2	Sequencing coverage	14.3 x
MIGS-30	Assemblers	Newbler, velvet
MIGS-32	Gene calling method	Prodigal 2.5
	INSDC ID	AGSE00000000
	GenBank Date of Release	13-MAY-2014
	GOLD ID	Gi07590
	NCBI project ID	PRJNA50621
	Database: IMG	2528311135
MIGS-13	Source material identifier	DSM 30083
	Project relevance	Tree of Life, GEBA

### Growth conditions and DNA isolation

A culture of strain DSM 30083^T^ was grown aerobically in DSMZ medium 1 [41] at 37°C. Genomic DNA was isolated using MasterPure Gram-Positive DNA Purification Kit (Epicentre MGP04100) following the standard protocol provided by the manufacturer but modified by incubation on ice over night on a shaker. DNA is available from DSMZ through the DNA Bank Network [53].

### Genome sequencing and assembly

The genome was sequenced using a combination of 454-GS-FLX-Titanium and Illumina GAii platforms. Illumina contigs of a length greater than 800 bp were shredded into pieces of up to 1000 bp at 200 bp intervals prior to the velvet [54] assembly. An additional round of automated gap closure yielded a draft version of the genome sequence comprising 37 contigs. Further gap closure *via* primer walking and finishing with Consed [55] was conducted at LGC Genomics (Berlin) and resulted in three aligned contigs for the chromosome and one for the plasmid.

### Genome annotation

Genes were identified using Prodigal [56] as part of the JGI genome annotation pipeline [57]. The predicted CDSs were translated and used to search the National Center for Biotechnology Information (NCBI) nonredundant database, UniProt, TIGR-Fam, Pfam, PRIAM, KEGG, COG, and InterPro databases. Identification of RNA genes were carried out by using HMMER 3.0rc1 [58] (rRNAs) and tRNAscan-SE 1.23 [59] (tRNAs). Other non-coding genes were predicted using INFERNAL 1.0.2 [60]. Additional gene prediction analysis and functional annotation was performed within the Integrated Microbial Genomes - Expert Review (IMG-ER) platform [61] CRISPR elements were detected using CRT [62] and PILER-CR [63].

## Genome properties

**Figure 3:**
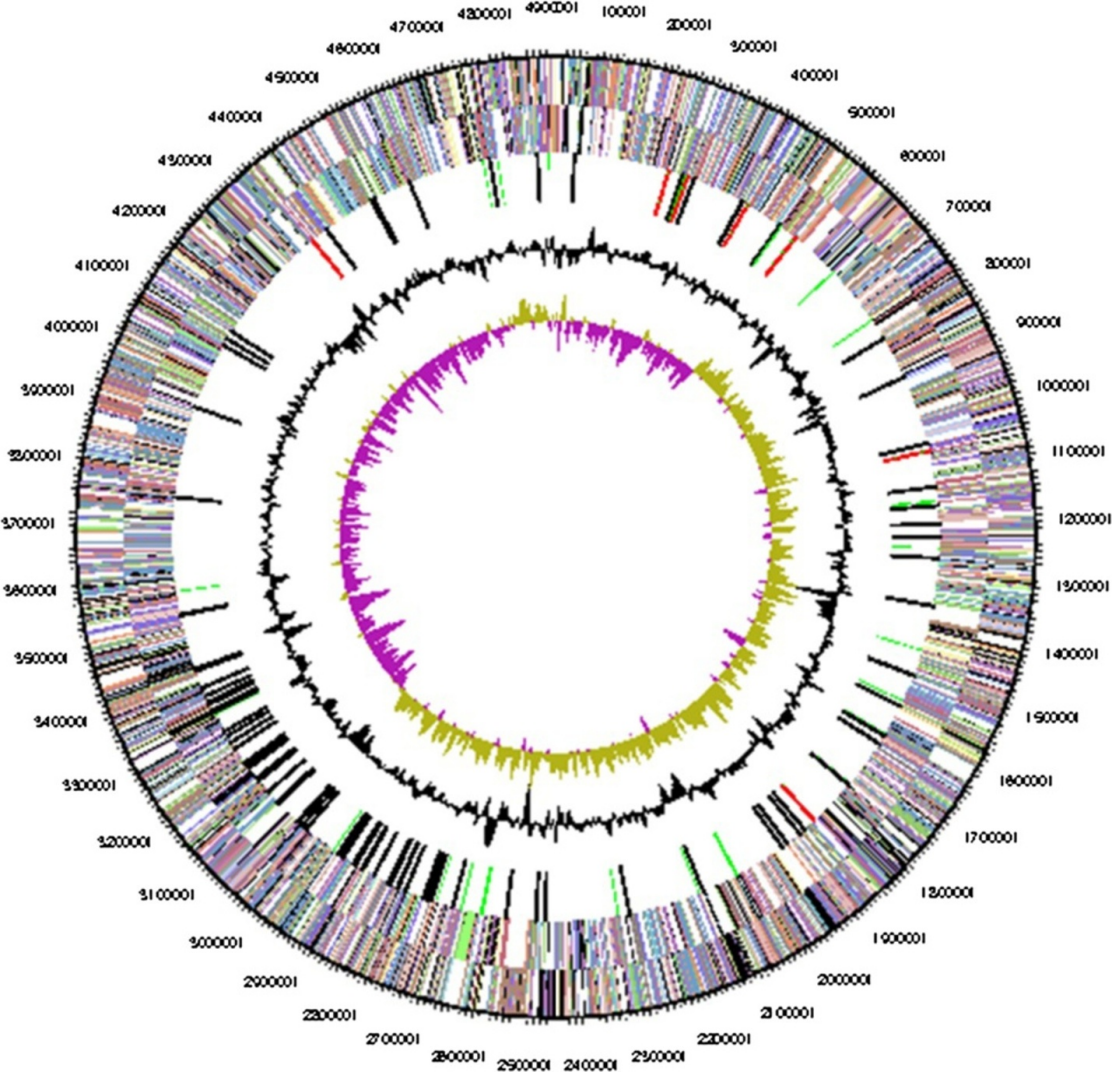
**Graphical map of the chromosome.** From bottom to the top: Genes on forward strand (colored by COG categories), Genes on reverse strand (colored by COG categories), RNA genes (tRNAs green, rRNAs red, other RNAs black), G+C content (black), G+C skew (purple/olive).

**Figure 4:**
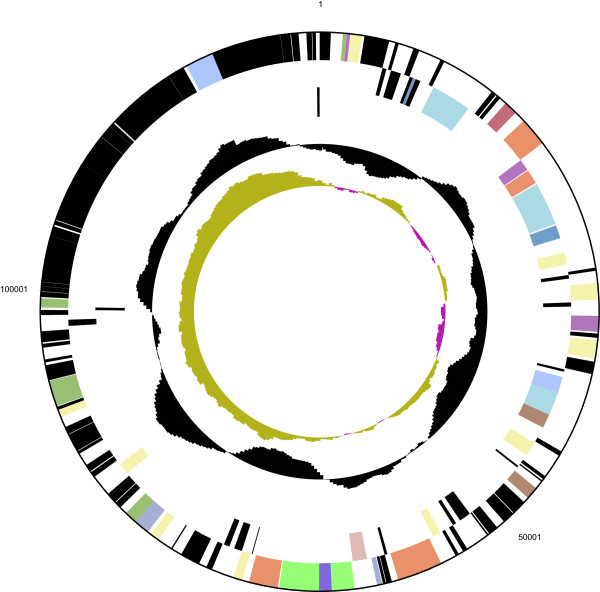
**Graphical map of the plasmid.** From bottom to the top: Genes on forward strand (colored by COG categories), Genes on reverse strand (colored by COG categories), RNA genes (tRNAs green, rRNAs red, other RNAs black), G+C content (black), G+C skew (purple/olive).

The genome statistics are provided in Table 3, Figure 3 and Figure 4. The genome of strain DSM 30083^T^ has a total length of 5,038,133 bp and a G+C content of 50.6%. Of the 4,937 genes predicted, 4,762 were identified as protein-coding genes, and 175 as RNAs. The majority of the protein-coding genes were assigned a putative function (84.2%) while the remaining ones were annotated as hypothetical proteins. The distribution of genes into COGs functional categories is presented in Table 4.

**Table 3:** **Genome statistics**

Attribute	Value	% of total
Genome size (bp)	5,038,133	100.0
DNA coding region (bp)	4,492,959	89.2
DNA G+C content (bp)	2,551,375	50.6
Number of scaffolds MIGS-9	2	
Extrachromosomal elements MIGS-10	1	
Total genes	4,937	100.0
RNA genes	175	3.5
rRNA operons	7	
tRNA genes	58	1.2
Protein-coding genes	4,762	96.5
Genes with function prediction (proteins)	4,157	84.2
Genes in paralog clusters	3,570	72.3
Genes assigned to COGs	3,651	74.0
Genes assigned Pfam domains	4,365	88.4
Genes with signal peptides	447	9.1
Genes with transmembrane helices	1,132	22.9
CRISPR repeats	2

**Table 4:** **Number of genes associated with the general COG functional categories**

Code	Value	% age	Description
J	182	4	Translation, ribosomal structure and biogenesis
A	2	1	RNA processing and modification
K	298	7	Transcription
L	197	5	Replication, recombination and repair
B	0	0	Chromatin structure and dynamics
D	35	1	Cell cycle control, cell division, chromosome partitioning
Y	0	0	Nuclear structure
V	50	1	Defense mechanisms
T	174	4	Signal transduction mechanisms
M	239	6	Cell wall/membrane/envelope biogenesis
N	114	3	Cell motility
Z	0	0	Cytoskeleton
W	1	0	Extracellular structures
U	137	4	Intracellular trafficking and secretion, and vesicular transport
O	137	3	Posttranslational modification, protein turnover, chaperones
C	276	7	Energy production and conversion
G	413	10	Carbohydrate transport and metabolism
E	359	8	Amino acid transport and metabolism
F	99	2	Nucleotide transport and metabolism
H	160	4	Coenzyme transport and metabolism
I	99	2	Lipid transport and metabolism
P	237	6	Inorganic ion transport and metabolism
Q	69	2	Secondary metabolites biosynthesis, transport and catabolism
R	426	11	General function prediction only
S	370	9	Function unknown
-	1286	26	Not in COGs

## Insights into the genome

### Which *E. coli* genomes actually represent *E. coli*?

Since the focus of this study is the *E. coli* type strain DSM 30083^T^, we will only discuss genomic aspects related to this strain in the following. Indeed, only the availability of the type-strain genome enables one to assess with modern genome sequence-based taxonomic methods whether or not the large number of genome-sequenced *E. coli* strains actually belong to this species. The taxonomist’s main criterion for species affiliation is the 70% DNA:DNA hybridization (DDH) similarity threshold [64, 65], but here we use an improved modern variant of the method, which is based on intergenomic sequence distances [24, 25]. This approach retains consistency with the microbial species concept because the traditional DDH is, on average, closely mimicked, but digital DDH (dDDH) avoids the pitfalls of traditional DDH due to the much lower error rate in genome sequencing [26].

**Figure 5:**
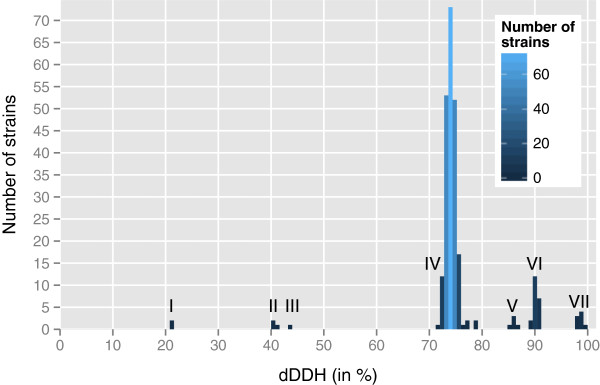
**Histogram of the digital DDH similarities between the type strain, DSM 30083**^**T**^ **, and other genome-sequenced** ***E. coli*** **strains as well as outgroups.** Interesting groups are marked by Roman numerals I-VII: *Escherichia hermannii* and *Shimwellia blattae* (I)*, E. fergusonii and E. albertii* (II)*, E.* sp. TW09308 (III), *E. coli* (IV-VII). Regarding the revised phylotypes from [66] (compare Figure 6), phylotype B2 is covered by dDDH groups V, VI, and VII with VII being the group containing (among other strains) type strain DSM 30083^T^ itself and its closest relative *E. coli* S88. IV marks the biggest group which includes phylotypes A, B1, D1, D2, D3, E, F1, F2 and *Shigella* I, IIa, IIb and III. The full list of dDDH values and affiliation to phylotypes is contained in Additional file 2.

Figure 5 shows the dDDH similarities between DSM 30083^T^ and a selection of 250 *E. coli* strains (see Additional file 2 for a full list) as well as outgroup genomes inferred using the Genome-to-Genome Distance Calculator [24], version 2 [25], which is based on the Genome BLAST Distance Phylogeny (GBDP) approach [24, 25]. Apparently, all strains identified as *E. coli* are within the 70% range of the type strain and hence need no reclassification (which would be the case for DDH values below the 70% threshold). The analysis also confirms that *Shigella* (within dDDH group IV) is placed within *E. coli*; this was already known from traditional DDH studies, yet the name *Shigella* was retained to not cause confusion in medical microbiology [39]. In accordance with the taxonomic classification, none of the strains from other *Escherichia* species yielded a dDDH similarity >70% (Figure 5).

For easing the comparison with literature data, we used the phylotypes suggested by [67–69] and revised according to the sixth picture in [66], which reassigned strains to phylotypes in most cases where it was necessary to render them monophyletic in a phylogenetic analysis of *E. coli* core genes (based on nucleotide alignments of 1,278 core-genes from 186 *E. coli* genomes). We had to additionally split phylotype D into D1, D2 and D3 because this phylotype actually was distributed over three distinct clades in [66], and for analogous reasons had to split F into F1 and F2 and *Shigella* II into *Shigella* IIa and *Shigella* IIb. The affiliation of the genomes present in our data set to the original phylotypes, if available, and the revised ones is contained in Additional file 2. The affiliations of *E. coli* strains to serovars were collected from GOLD [1], those to pathovars from [1] and [70]; they are also listed in the supplement.

Regarding the dDDH groups V, VI and VII in Figure 5 containing the *E. coli* strains with a dDDH similarity to the type strain of around 85% or higher, those with an assigned revised phylotype uniformly belonged to phylotype B1. A histogram depicting the dDDH similarities between all strains used in this study is contained in Additional file 1.

### Phylogenetic analysis with nucleotide GBDP

**Figure 6:**
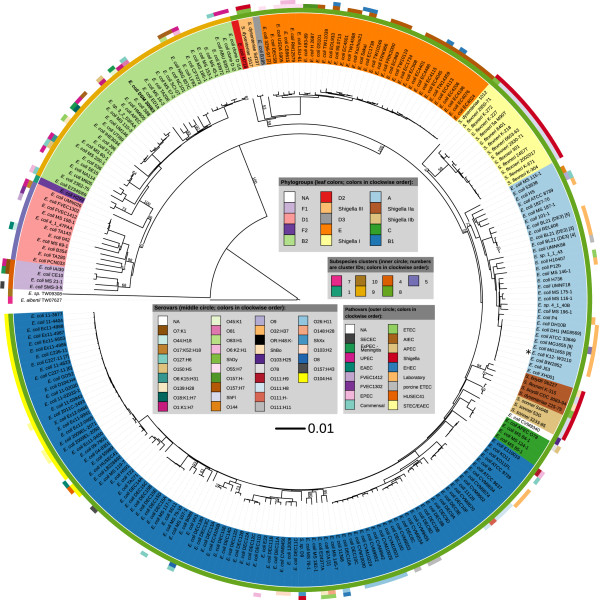
**Whole-genome phylogeny inferred using the latest GBDP version** [25] **and rooted with Escherichia albertii.** Other outgroup organisms, separated by long branches, were removed to ease visualization (*E. hermannii*, *Shimwellia blattae*, and *E. fergusonii*) but are shown in Additional file 1. Numbers above branches are greedy-with-trimming pseudo-bootstrap [73] support values from 100 replicates if larger than 50%. Leaves are colored according to their affiliation to phylotypes. The outer circles show the affiliation of the strains to potential subspecies, pathovars and serovars (if the information was retrievable). Labels with numbers in square brackets are duplicates (due to label shortening) and refer to the following full strains/GenBank accessions: [1] CS6:LT+:ST+, [2] TW07815, [3] AM946981, [4] CP001509, [5] CP001665, [6] AFST00000000, [7] AFRH00000000, [8] K-12, MG1655 U00096, and [9] CM000960. An asterisk (*) indicates the K-12 wild type. ITOL [74] was used to visualize the tree inferred using FastME [75].

Figure 6 depicts a phylogenetic tree of the same strains inferred using GBDP, the highly reliable method [71] to calculate intergenomic distances, on which the inference of digital DDH values as shown in Figure 6 is also based [24, 25]. The branch support values in this tree (Figure 6) originate from pseudo-bootstrapping [25], a procedure which is known as conservative [72] and in the context of GBDP tends to underestimate branch support particularly for branches close to the tips [73]. Accordingly, the tree shows a well-supported backbone whereas terminal branches reveal less support.

Nevertheless, the tree topology (Figure 6) shows all revised phylotypes as monophyletic, and some of them with high support. According to Figure 6 the type strain DSM 30083^T^ is placed within phylotype B2 with *E. coli* S88 as its closest neighbor. The observation that the *Shigella* phylotypes occur in three different clades, but that these are all positioned within *E. coli*, together with earlier studies [76, 77] provides evidence against a recent study [78] which proposes *Shigella* spp. as a sister group of *E. coli* rather than at least one of its subgroups. A possible reason might be that [78] utilized an alignment-free genome signature (“CVTree”) approach which was recently shown to be less accurate than GBDP [71]. High (92%) support was achieved for a clade comprising phylotypes A, B1, C, *Shigella* I, *Shigella* IIa and *Shigella* IIb, and maximum support for a parent clade of that clade, also comprising phylotypes D2, D3, E and *Shigella* III. The serovars and pathovars, as far as attributable to the genomes used in this study, showed lower agreement with the tree topology. This might be due to the highly diverse adaptive paths present in *E. coli* [77].

### Phylogenetic analysis of proteome sequences

The genome sequences of a subset of 50 representative genome-sequenced strains were phylogenetically investigated in a complementary analysis using the DSMZ phylogenomics pipeline as previously described [79–86] using NCBI BLAST [87], OrthoMCL [88], MUSCLE [89], RASCAL [90], GBLOCKS [91] and MARE [92] to generate concatenated alignments of distinct selections of genes (supermatrices). Maximum likelihood (ML) [93] and maximum parsimony (MP) [94, 95] trees were inferred from the data matrices with RAxML [96, 97] and PAUP* [98], respectively, as previously described [79–86].

**Figure 7:**
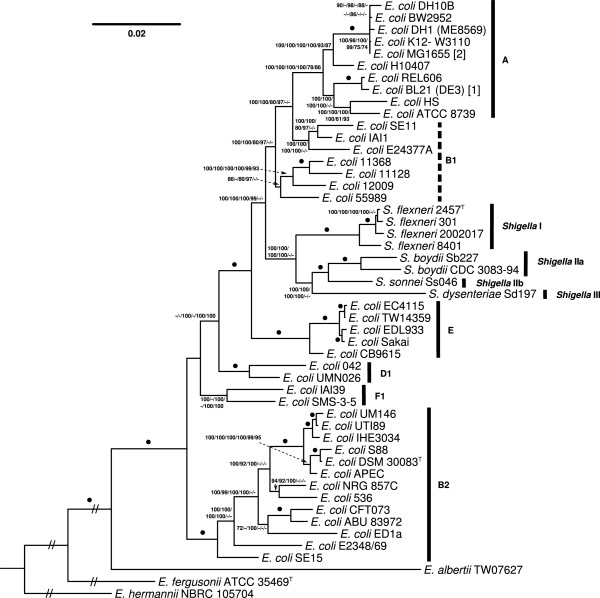
**Phylogenetic tree inferred from the MARE-filtered supermatrix under the maximum likelihood (ML) criterion and rooted with** ***E. hermannii*** **NBRC 105704.** Branch lengths within the outgroup were shortened to improve visualization. The branches are scaled in terms of the expected number of substitutions per site. Numbers above / below the branches (from left to right) are bootstrapping support values (if larger than 60%) from (i) ML MARE-filtered supermatrix; (ii) maximum-parsimony (MP) MARE-filtered supermatrix; (iii) ML “full” supermatrix, (iv) MP “full” supermatrix, (v) ML core-genes; (vi) MP core-genes analysis. Dots indicate branches with maximum support under all settings. Numbers in square brackets refer to further strain information as listed in the caption of Figure 6.

The topology of the ML MARE-filtered supermatrix analysis is shown in Figure 7 together with ML and MP bootstrap support values from all supermatrix analyses if larger than 60%. Support was maximum (100%) for the majority of branches under ML and MP (Figure 7). Again, in this tree all phylotypes are represented as monophyla with the sole exception of B1, which was revealed only in the core-gene analysis, much like in [66]. A further difference to the 2012 study [66] and the GBDP tree (Figure 6) is that *Shigella* phylotypes I, IIa, IIb and III formed a clade together; again this clade was not visible in the core-gene tree. In our view, trying distinct ways to generate supermatrices has the strong advantage that branches that are sensitive to gene selection can be revealed [79–86]. Whereas the above-mentioned groups are instable in this respect, others such as the group comprising phylotypes A, B1, E and all *Shigella* strains yield maximum support under all assessed gene selections; this large clade also obtained 100% support with GBDP (Figure 6). Average branch support under ML and MP, respectively, was 91.72/87.62% using the core genes only (101,755 variable, 21,474 parsimony-informative characters), 94.04/97.64% using the MARE-filtered supermatrix (285,814/99,071) and 90.3/97.49% using the entire supermatrix (456,246/153,146). This is largely in agreement with the tendency observed in previous studies using the same phylogenomics pipeline that more characters simply yield higher support, despite the frequent concerns regarding horizontal gene transfer [99], but might also indicate advantages of the removal of phylogenetically uninformative genes with MARE [92].

### Phylogenetic analysis of gene and ortholog content

**Figure 8:**
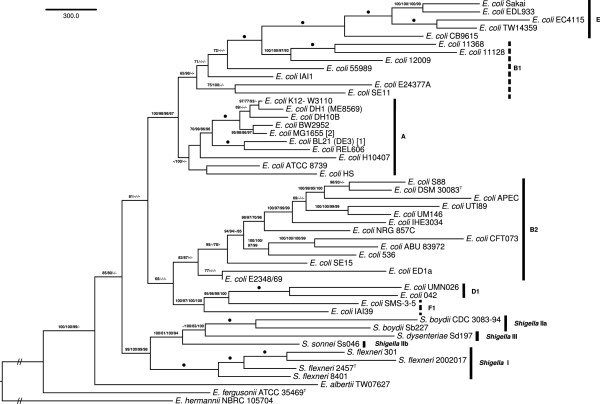
**Phylogeny inferred from the ortholog-content matrix under the maximum parsimony (MP) criterion and rooted with** ***E. hermannii*** **NBRC 105704.** The branches are scaled in terms of the minimum number of substitutions (DELTRAN optimization). Numbers above/below the branches (from left to right) are bootstrapping support values (if larger than 60%) from (i) MP ortholog-content matrix; (ii) maximum-likelihood (ML) ortholog-content matrix; (iii) MP gene-content matrix; (iv) ML gene-content matrix analysis. Dots indicate branches with maximum support under all settings.

The clusters of orthologs as inferred with OrthoMCL, as well as clusters of homologs inferred using re-implementation of the TribeMCL [100] algorithm as previously described [79–86], were converted to presence-absence matrices for phylogenetic inference using ML and MP. The topology of the MP ortholog-content analysis is shown in Figure 8 together with MP and ML bootstrap support values from ortholog-content and gene-content analyses if larger than 60%. In contrast to the GBDP (Figure 6) and supermatrix (Figure 7) analyses, *E. coli* forms a sister group of *Shigella* spp., but with at most moderate (80%) support. Similarly, the clade containing both is at most moderately supported. Support for a monophyletic *Shigella* is high, however (98-100%). The phylotypes are revealed as monophyletic except for F1 and B1 (with strong support against them forming a clade, respectively).

### The within-species difference of genomic G+C content

**Figure 9:**
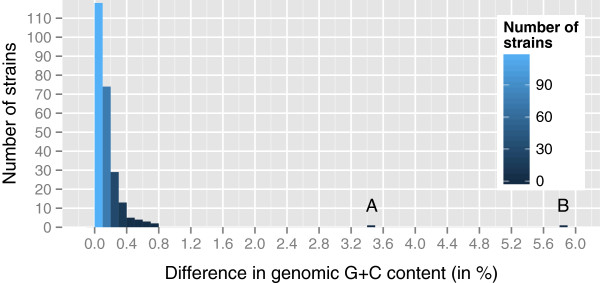
**Histogram of the differences in genomic G+C content between the** ***E. coli*** **type strain and the other 250 strains contained in the data set.** In accordance to a within-species difference of at most 1% in the G+C content [26], none of the differences between the distinct strains of *E. coli* are above that threshold. The G+C differences to *E. hermannii* NBRC 105704 (3.4%, “A”) and *Shimwellia blattae* DSM 4481 (5.9%, “B”) are considerably larger, whereas *E. albertii*, *E. fergusonii* and “*Escherichia* sp. TW09308” (which are also phylogenetically more close to *E. coli*; see Figure 6 and Figure 7) cannot be distinguished from *E. coli* using the G+C content.

The G+C content of 50.6% inferred from the genome sequence is in agreement with the value of 50.7 ± 0.6 mol% determined for strain DSM 30083^T^ by Albuquerque et al. [101], but differs slightly from the G+C content of 51.0-51.7 mol%, determined from deposit ATCC 11775^T^ [29]. The G+C content range of *E. coli* strains was reported as 48.5-52.1 mol% [29], in conflict with more recent results [26]. Thus affiliation to *E. coli* was also assessed by calculating the genomic G+C content of all 251 strains in the data set and the difference to the G+C content of the type strain, DSM 30083^T^. Results shown in Figure 9 are in agreement with the result from [26] that within-species differences in the G+C content are almost exclusively below 1%. As expected, *E. coli* cannot be distinguished from the other *Escherichia* species based on G+C content.

### The 131-kb plasmid of *E. coli* DSM 30083^T^

The *E. coli* type strain DSM 30083^T^ contains a single circular incFII-type plasmid with a size of 131,289 bp and a G+C content of 49.3% (Figure 4). A homologous plasmid that just exhibits an inversion of 15 kb and an indel (insertion/deletion) of 3 kb is present in the closest relative *E. coli* strain S88 (CU928146). The 131-kb plasmid harbors a type IV secretion system and a highly syntenous conjugative plasmid has been identified in a multidrug-resistent *Salmonella enterica* strain CVM29188 (NC_011076) [102] thus providing strong evidence of natural interspecies exchange of the extrachromosomal element.

### Physiological discrimination of *E. coli* DSM 30083^T^and DSM 18039

Since the genomes of both *E. coli* strains DSM 30083^T^ and K-12 MG1655 (=DSM 18039) fall into strongly separated clusters, the question of phenotypic differences between the type strain and the widely used laboratory strain arises, too. We thus also investigated the substrate spectrum of using PM-01 and PM-02 microplates as described above (see also Additional file 1). In contrast to DSM 30083^T^, DSM 18039 was positive for dulcitol, D-xylose, α-keto-glutaric acid, *m-* tartaric acid, α-hydroxy-butyric acid, 5-keto-d-gluconic acid, but negative for l-glutamic acid, d-glucosaminic acid, tween 20, tween 40, mono-methyl succinate, *N*-acetyl-d-galactosamine, d-arabinose, d-raffinose, l-sorbose, d-tagatose. On API 20E strips (see Additional file 1) strain DSM 18039^T^ in contrast to *E. coli* 30083^T^ was negative for l-ornithine. A unique diagnostic trait of all completely sequenced K-12 strains that allow the discrimination from other *E. coli* isolates is a deletion of 3,205 bp in the *aga* gene cluster that is required for the conversion of *N*-acetyl-d-galactosamine [103].

### Subdivision of *E. coli* revisited

As shown above, after a small number of revisions as conducted in [66] and partially in this study, the proposed phylotypes of *E. coli* appear monophyletic in the phylogenetic analyses of genome-scale data. The sole exception is phylotype B1, whose monophyly is confirmed in Figure 6 but shows a sensitivity to gene selection in analyses of proteome sequences (Figure 7). The additional question arises, however, whether or not the phylotypes are not only monophyletic but also are comparable to each other with respect to the level of character divergence within each group. This would be advantageous for (formal or informal) classification, as can easily be shown by a comparison with the 70% DDH rule for delineating bacterial species. There is, unfortunately, no guarantee that the set of strains in the 70% (d)DDH range of a type strain form a monophyletic group unless the distances are ultrametric [26]. But on the other hand, in contrast to the monophyly criterion itself, the consequent application of the 70% DDH rule by construction yields groups with a similar upper bound of character divergence. The same reasoning also holds for organisms not covered by the Bacteriological Code. For instance, whereas birds, mammals and primates are all monophyletic according to current knowledge, comparing birds and mammals regarding, say, species numbers makes much more sense than comparing birds and primates.

To assess the homogeneity of the revised *E. coli* phylotypes, some of their cluster statistics were calculated with OPTSIL [104] version 1.5 and the matrix of intergenomic distances used for inferring dDDH values (Figure 5). Average within-cluster distances ranged between 0.00098 and 0.01571 with a median of 0.00503, whereas maximum within-cluster distances ranged between 0.00121 and 0.02199 with a median of 0.01444. Further, clustering optimization as implemented in OPTSIL was conducted using the revised phylotypes as reference partition; details are found in Additional file 3. The maximum agreement with the reference partition was obtained for a combination of clustering parameters that yielded 32 clusters, way more than the number of phylotypes plus outgroups that were input into clustering optimization.

This analysis shows that the phylotypes of *E. coli*, even if revised to obtain monophyly of all phylotypes in the phylogenetic analyses of genome-scale data as conducted in [66] and this study (Figure 6), are not homogeneous regarding their divergence as measured using genome-scale nucleotide data. This can also be shown indirectly by comparing the phylotypes to a clustering conducted with the slightly higher distance threshold of 0.0242, which corresponds to 79.3% dDDH. The tree in Figure 6 is annotated with this clustering, too; it yields five clusters, four of which obtain GBDP pseudo-bootstrap values between 98% and 100%. Four of these clusters directly correspond to one phylotype, respectively, namely B2, D1, F1 and F2, whereas the fifth cluster comprises all remaining phylotypes, including all *Shigella* spp. (Figure 6). Interestingly, in contrast to some phylotypes, this cluster is supported in proteome-based trees under all investigated settings (Figure 7). It is not supported by the gene-content based phylogenies (Figure 8), but these neither yield support against this cluster. Thus if measured from genome-scale nucleotide data the phylotypes B2, D1, F1 and F2, as well as the combination of all remaining clusters have approximately the same level of divergence, respectively.

### Delineation of subspecies revisited

Bacterial subspecies were traditionally not determined based on a distance or similarity threshold, but on a qualitative assessment of few selected phenotypic characters [65, 105, 106]. A quotation from [64] is worth reproducing here: “Subspecies designations can be used for genetically close organisms that diverge in phenotype. There is some evidence, based on frequency distribution of Δ*T*_m_ values in DNA hybridization, that the subspecies concept is phylogenetically valid. (…) There is a need for further guidelines for designation of subspecies.” Particularly because the availability of complete genome sequences allows for the transition to genome-based taxonomy, yielding to a considerable increase in phylogenetic resolution [99], rules for a genome-based, quantitative approach to subspecies delineation in analogy to the 70% (d)DDH threshold for the delineation of species [24, 25, 65], would be desirable.

However, as emphasized in [26], inconsistencies can occur when distance or similarity thresholds are used and the underlying distances specifically deviate from ultrametricity. These potential pitfalls are a general consequence of the direct use of pairwise distances or similarities (which is not a phylogenetic method) for assessing taxonomic affiliations [104] and not directly related to traditional or digital DDH. Fewer taxonomic problems are expected when comparisons between two non-type strains are avoided (which is necessary for reasons of nomenclature anyway), but this does not entirely prevent pitfalls [26]. Nevertheless, whether paradoxes really occur in practice depends on the distance threshold and the specific deviation of the data under study from the ultrametric condition [26]. Hence, if a threshold for delineating bacterial subspecies is of interest, it makes sense to choose it so as to minimize the potential of taxonomic inconsistencies related to non-ultrametric data as far as possible. This can be done for bacterial subspecies precisely because by tradition they have not been determined based on a distance or similarity threshold, in contrast to the species rank, hence such a threshold can now be carefully chosen based on the above-mentioned principles.

Using the *E. coli* data as starting point, augmented by the data set used in [26] containing completely sequenced genomes for 105 genera of *Archaea* and *Bacteria*, in addition to criteria from the literature we have devised a criterion called “clustering consistency” for optimizing thresholds for sub-specific bacterial lineages. Compared to the analysis of frequency distributions of (d)DDH values as mentioned in [99], this approach has the advantage that it directly addresses how to best cluster the sequences. The analyses described in detail in Additional file 3 show that regarding within-species clustering consistency a distance threshold corresponding to 79-80% dDDH makes most sense for both the *E. coli* and the 105-genera *Archaea* and *Bacteria* data sets. In addition to clustering consistency, a value around 80% has a couple of other advantages. For instance, it is sufficiently larger than the species boundary at 70% but nevertheless does not yield too many subspecies if applied strictly. This is particularly important regarding the low number of currently described subspecies in the literature, which in our view makes it also impossible to estimate dDDH subspecies boundaries from the currently validly named subspecies. Furthermore, values between 90% and 95% dDDH could be reserved in the future for taxonomic ranks such as “variety”. Finally, values approaching 100% are unsuitable because they might represent distinct clones or deposits of the same strain or even genome sequences obtained several times from the same strain.

### Taxonomic consequences for *E. coli*?

As mentioned above, *E. coli* is an attractive example for the application of the 79-80% dDDH rule (Figure 6). Hence, the description of subspecies of *E. coli* is the next logical consequence. Regarding practice, it is noteworthy that the already established detection of phylotypes [67–69] will help detecting the subspecies, too, because the (revised) phylotypes are either identical to subspecies or to subsets of subspecies (Figure 6). Furthermore, even incompletely sequenced genomes can be used to detect the subspecies by the comparison with the type strains using the GGDC server [24, 25]. Apparently, *Shigella* spp. would not only be placed within *E. coli* [107] but even embedded within one of the subspecies defined at the 79-80% dDDH boundary (Figure 6). Crucially, this changes nothing regarding the status of *Shigella*: if this name is to be retained not to cause confusion in medical microbiology anyway [39], it simply does not matter whether or not it otherwise would be placed entirely within *E. coli* or even entirely within a yet to be established subspecies of *E. coli*.

However, the placement of *Shigella* yields yet another problem for the division of *E. coli* into subspecies. An approach to describe subspecies for *E. coli* could start with the largest cluster in Figure 6, which contains most of the genome-sequenced strains including strain K-12, but also all strains of *Shigella*. Following the guidelines of the *Bacteriological Code* (1990 revision) [2] the type strain of this subspecies would be strain Newcastle^T^ (=NCTC 4837^T^) representing *E. coli* subsp. *dysenteriae* (Shiga 1897) Castellani and Chalmers 1919*,* with strain U5/41^T^ automatically becoming the type strain of *E. coli* subsp. c*oli* (Shiga 1897) Castellani and Chalmers 1919*.* Thus establishing this subspecies of *E. coli* would taxonomically conflict with the purpose of retaining *Shigella* [39], hence we refrain from proposing taxonomic consequences here. The dDDH boundary suggested in this study for delineating subspecies might nevertheless be of use on many other groups of *Bacteria* and *Archaea* that are not hampered by similar (taxonomic) constraints.

## Conclusions

This study presents the genome sequence for the *E. coli* type strain DSM 30083T, whose marked physiological and genomic differences from the model bacterium *E. coli* K-12 are reviewed in detail. A phylogenomic analysis of 250 *E. coli* strains reveals that their arrangement into the phylotypes suggested in the literature, even though they mostly appear monophyletic, does not yield a uniform level of character divergence. We thus propose an alternative arrangement and discuss it in the context of the subspecies rank. This is of special interest because bacterial subspecies were traditionally not determined based on a distance or similarity threshold but an approach to quantitatively delineate them has been requested in the literature. Based on an investigation of genome-sequenced strains from > 100 genera, including *E. coli*, and the criterion of clustering consistency, we suggest a boundary of 79-80% dDDH for delineating subspecies within *Bacteria* and *Archaea*. Such dDDH-based subspecies delineation is available via the GGDC web service.

In *E. coli*, the criterion yields five subspecies, one of which includes strain 30083T and is identical to phylogroup B2. Strain K-12, together with *Shigella* and the majority of *E. coli* strains, belongs to another subspecies. Issues of nomenclature prevent taxonomic consequences in *E. coli*, but the methodology applied here is of general interest for bacterial subspecies delineation.

## Electronic supplementary material

Additional file 1: **Supplementary figures.** (PDF )

Additional file 2: **List of** ***E. coli*** **genomes used in this study.** (XLS )

Additional file 3: **Delineating bacterial subspecies.** (PDF )
