# Supplementary material for: Complete genome sequence of DSM 30083T, the type strain (U5/41T) of Escherichia coli, and a proposal for delineating subspecies in microbial taxonomy
Source: Stand Genomic Sci. 2014 Dec 8;9:2. doi: 10.1186/1944-3277-9-2 (PMC4334874; doi:10.1186/1944-3277-9-2)
Supplement: Supplementary file 1 — Additional file 1: Supplementary figures. (PDF ) [file 40793_2014_2_MOESM1_ESM.pdf]

Supplementary figures

Additional file 1 to: "Complete genome sequence of DSM 30083^{T}, the type strain (U5/41^{T}) of *Escherichia coli*, and a proposal for delineating subspecies in microbial taxonomy"

Authors: Jan P. Meier-Kolthoff, Richard L. Hahnke, Jörn Petersen, Carmen Scheuner, Victoria Michael, Anne Fiebig, Christina Rohde, Manfred Rohde, Berthold Fartmann, Lynne A. Goodwin, Olga Chertkov, Tatiparthi B. Reddy, Amrita Pati, Natalia N. Ivanova, Victor Markowitz, Nikos C. Kyrpides, Tanja Woyke, Markus Göker*, Hans-Peter Klenk

***Corresponding author:** Markus Göker <markus.goeker@dsmz.de>

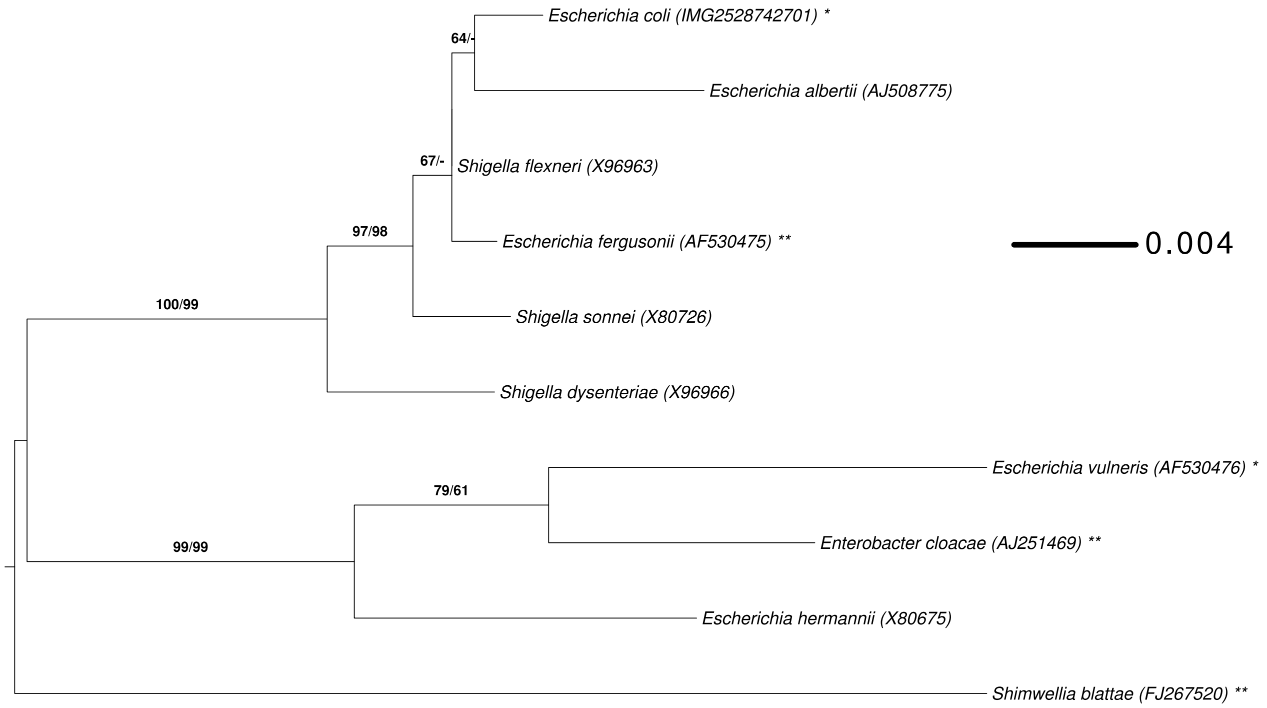

Figure S1 - 1: Phylogenetic tree highlighting the position of *E. coli* relative to the type strains of the other species within the genus *Escherichia*. The tree was inferred from 1,433 aligned characters of the 16S rRNA gene sequence under the maximum likelihood (ML) criterion as previously described (Göker et al. 2011). *Shimwellia blattae* was included in the data set for use as outgroup taxon. The branches are scaled in terms of the expected number of substitutions per site. Numbers adjacent to the branches are support values from 1,000 ML bootstrap replicates (left) and from 1,000 maximum-parsimony bootstrap replicates (right) if larger than 60% (Göker et al. 2011). Lineages with type strain genome sequencing projects registered in GOLD (Pagani et al. 2012) are labeled with one asterisk, those also listed as 'Complete and Published' with two asterisks.

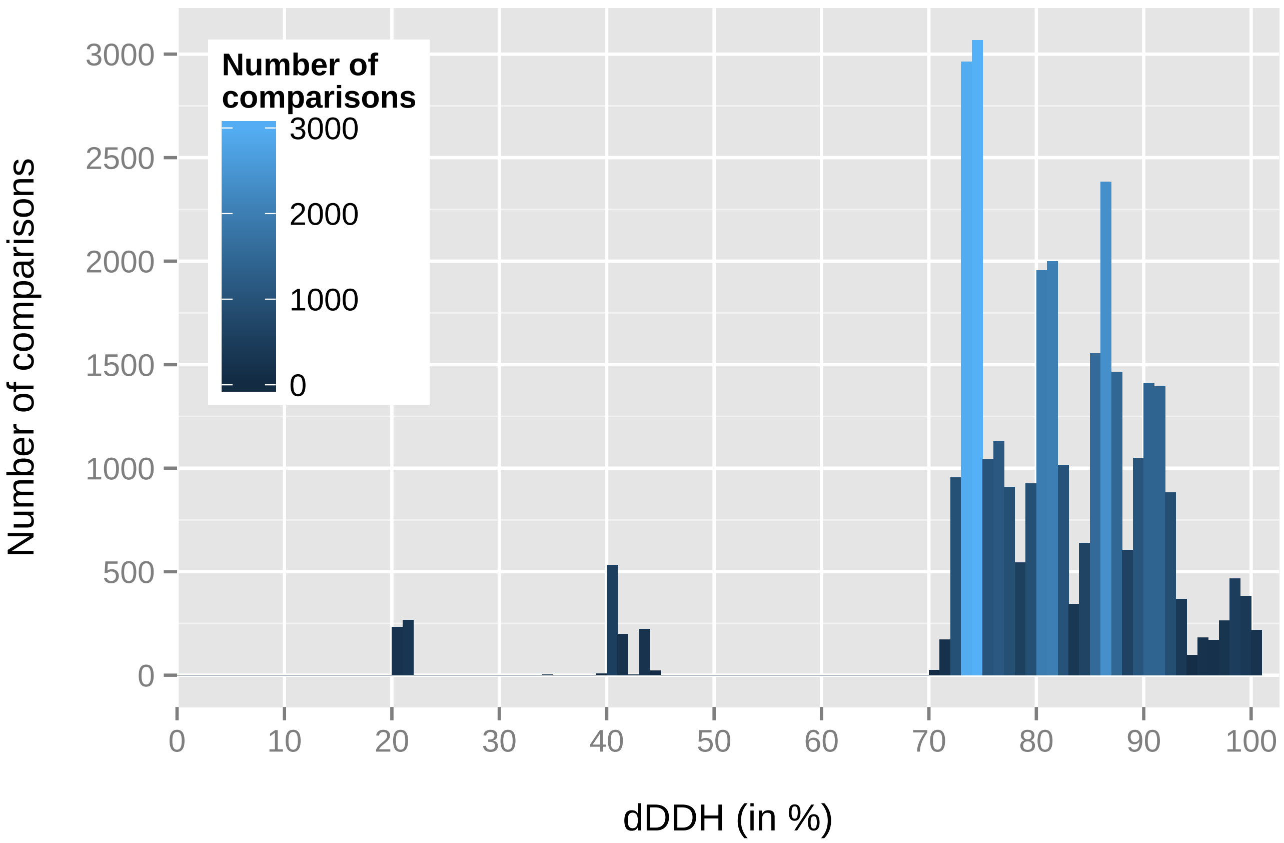

Figure S1 - 2: Histogram of all pairwise digital DDH similarities between all pairs of strains contained in the data set (see Supplement File S2).

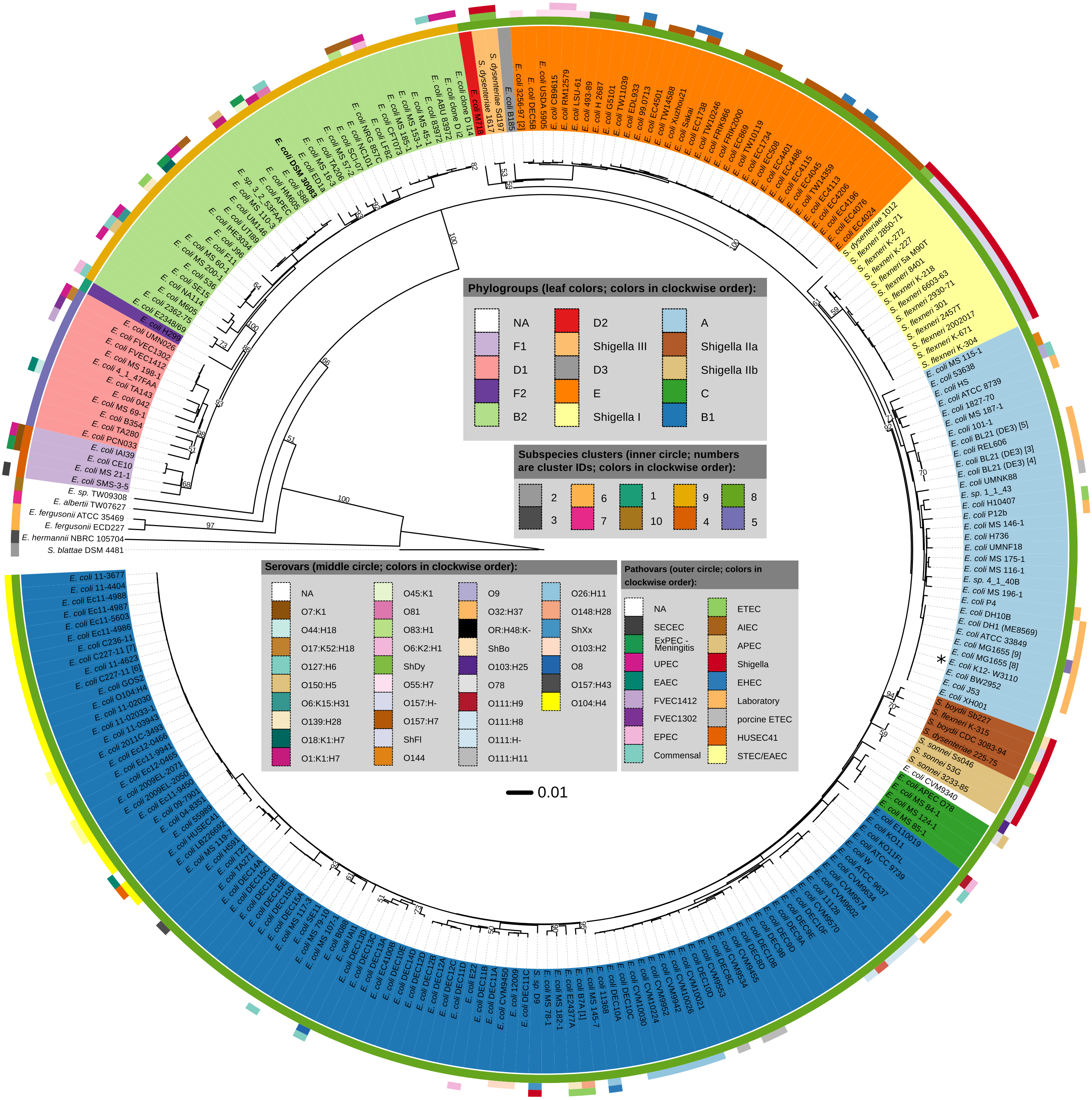

Figure S1 - 3: Whole-genome phylogeny inferred using the latest GBDP version (Meier-Kolthoff et al. 2013) and rooted with *Shimwellia blattae*. The only difference between this tree and the one shown in Figure 7 of the main manuscript is that here all outgroup organisms are shown: *Escherichia albertii*, *Escherichia hermannii*, *Shimwellia blattae*, and *Escherichia fergusonii*). Refer to Figure 7 of the main manuscript for further information.

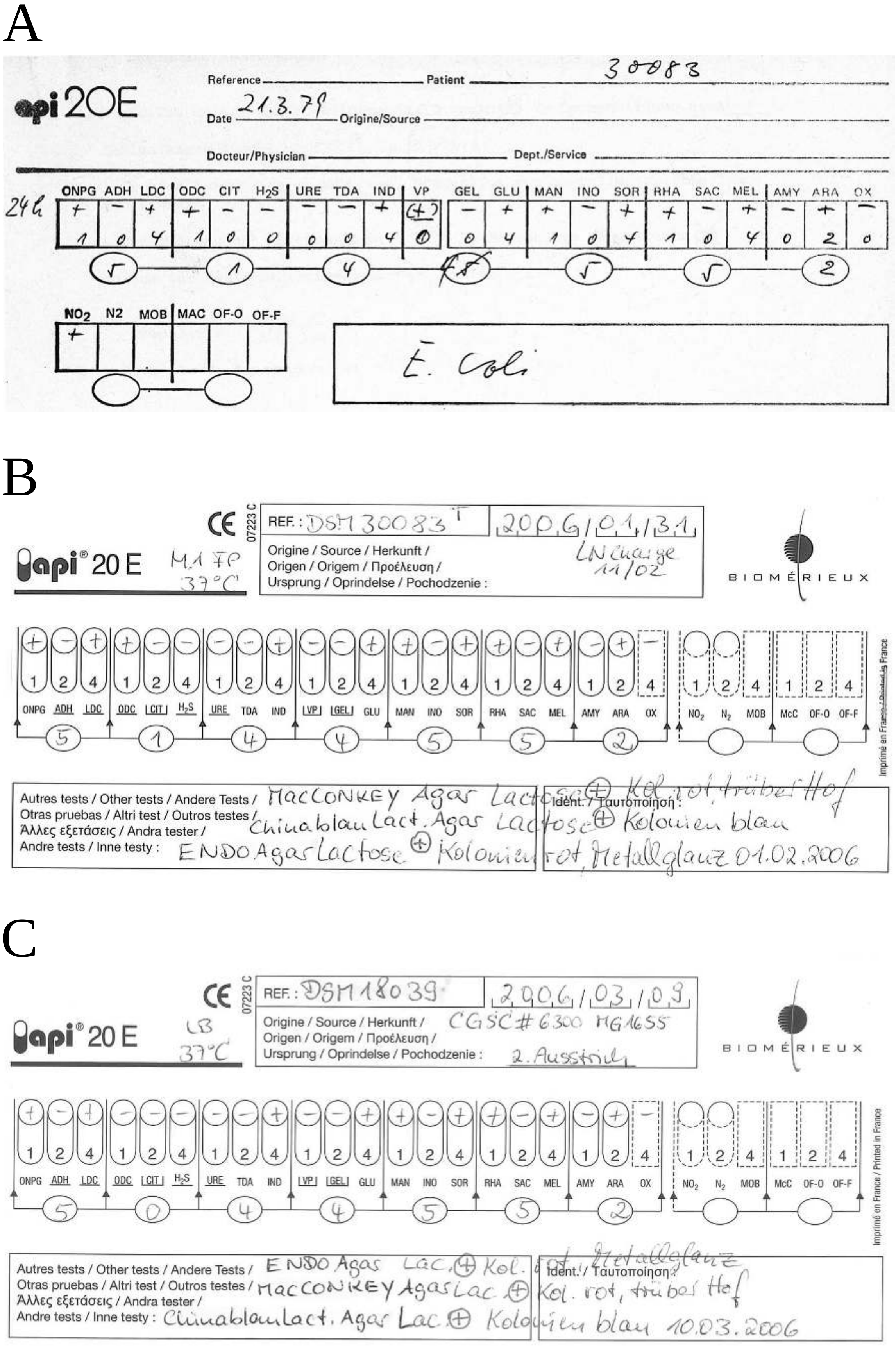

Figure S1 - 4: API 20E test of *E. coli* strains DSM 30083^{T} and DSM 18039. At the DSMZ, strain DSM 30083^{T} was tested for the first time with API 20E test stripes at 37°C for 24 h in 1979 (A), and additionally on McCognay agar, chinablue and ENDO agar (B). Strain DSM 18039 differed from DSM 30083^{T} in being positive for ornithine decarboxylase activity (C). Abbreviations of substrates are listed in the manufacturer protocol (bioMérieux, Nürtingen, Germany).

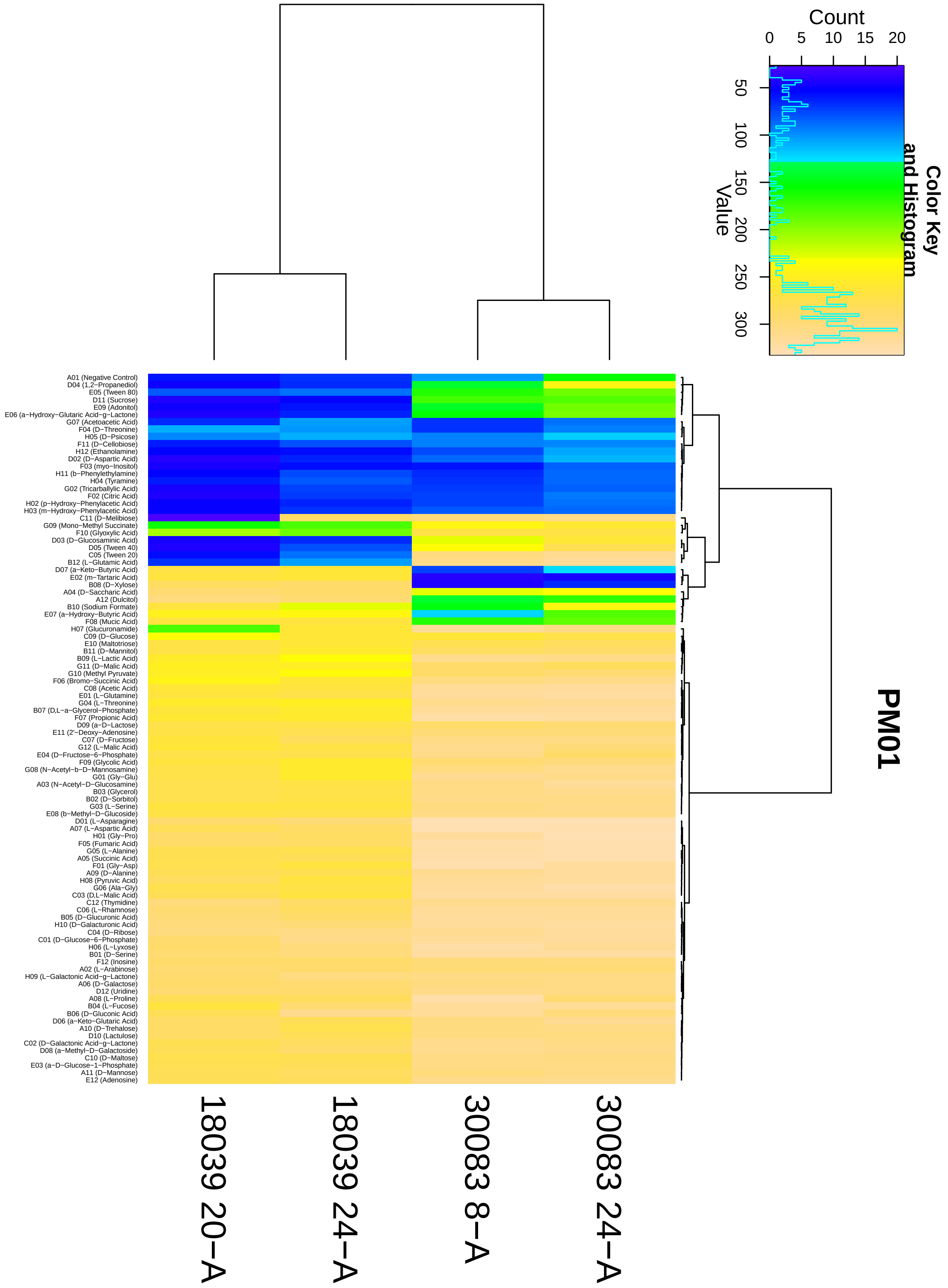

Figure S1 - 5: Heat map of the phenotype microarray results, microplate PM-01, for the type strain of *E. coli* compared to strain DSM 18039. Data were analysed with OPM version 1.1.2, using the maximum curve height parameter for plotting, inferred using smoothing splines.

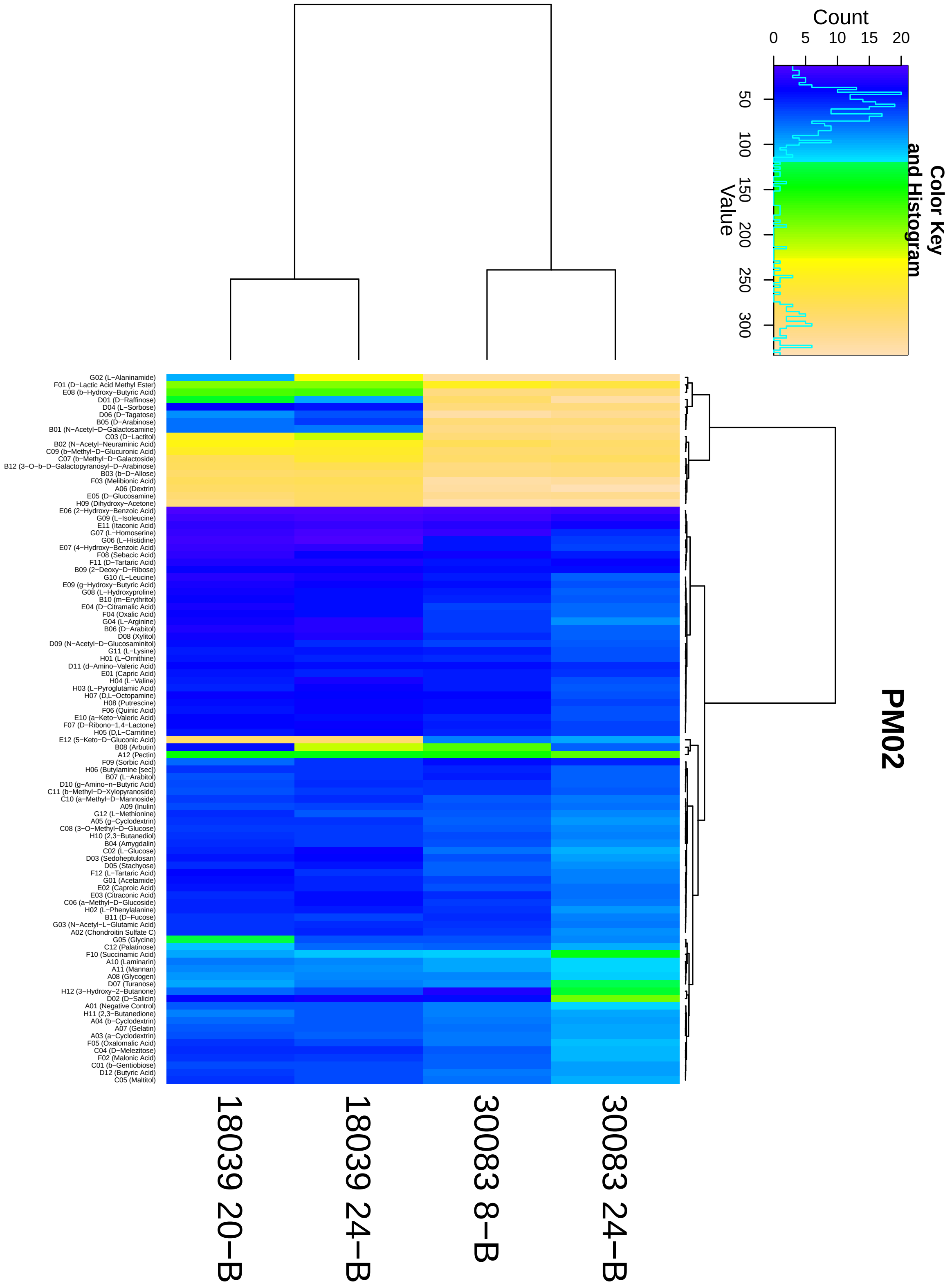

Figure S1 - 6: Heat map of the phenotype microarray results, microplate PM-02, for the type strain of *E. coli* compared to strain DSM 18039. Data were analysed with OPM version 1.1.2, using the maximum curve height parameter for plotting, inferred using smoothing splines.

References

Göker M, Cleland D, Saunders E, Lapidus A, Nolan M, Lucas S, Hammon N, Deshpande S, Cheng JF, Tapia R, et al. Complete genome sequence of *Isosphaera pallida* type strain ($IS1B^T$). Stand Genomic Sci 2011; 4:63-71.

Pagani I, Liolios K, Jansson J, Chen IM, Smirnova T, Nosrat B, Markowitz VM, Kyrpides NC. The Genomes OnLine Database (GOLD) v.4: status of genomic and metagenomic projects and their associated metadata. Nucleic Acids Res 2012; 40:D571-579.

Meier-Kolthoff JP, Auch AF, Klenk H-P, Göker M. Genome sequence-based species delimitation with confidence intervals and improved distance functions. BMC Bioinformatics 2013; 14: 60, (doi:10.1186/1471-2105-14-60).
