# Supplementary material for: Complete genome sequence of DSM 30083T, the type strain (U5/41T) of Escherichia coli, and a proposal for delineating subspecies in microbial taxonomy
Source: Stand Genomic Sci. 2014 Dec 8;9:2. doi: 10.1186/1944-3277-9-2 (PMC4334874; doi:10.1186/1944-3277-9-2)
Supplement: Supplementary file 3 — Additional file 3: Delineating bacterial subspecies. (PDF ) [file 40793_2014_2_MOESM3_ESM.pdf]

Delineating bacterial subspecies

Additional file 3 to: "Complete genome sequence of DSM 30083^{T}, the type strain (U5/41^{T}) of *Escherichia coli*, and a proposal for delineating subspecies in microbial taxonomy"

Authors: Jan P. Meier-Kolthoff, Richard L. Hahnke, Jörn Petersen, Carmen Scheuner, Victoria Michael, Anne Fiebig, Christina Rohde, Manfred Rohde, Berthold Fartmann, Lynne A. Goodwin, Olga Chertkov, T.B.K. Reddy, Amrita Pati, Natalia N. Ivanova, Victor Markowitz, Nikos C. Kyrpides, Tanja Woyke, Markus Göker*, Hans-Peter Klenk

***Corresponding author:** Markus Göker <markus.goeker@dsmz.de>

Introduction

We here describe our approach to finding clustering parameters suitable for delineating sub-specific bacterial lineages using the same intergenomic distances that can be used to estimate digital DNA:DNA hybridization values (Meier-Kolthoff et al. 2013, 2014). A main issue to be taken into account here is that clustering algorithms such as single or complete linkage clustering make no use of types such as type strains (in bacterial taxonomy) or type specimens (in zoology or botany). In contrast, taxonomic assessments such as the establishment of a new species can ultimately only be assessed by comparing with types. For instance, the 70% (d)DDH rule makes only sense if applied to the comparison of two type strains or of one non-type strain with a type strain (Meier-Kolthoff et al. 2014).

Meier-Kolthoff et al. (2014) have emphasized a number of pitfalls that are related to the use of pairwise distances or similarities for assessing taxonomic affiliations in general. These can occur because biological distances are not necessarily ultrametric. The magnitude of the problems depends on the chosen distance threshold T and whether or not, and if so in which way, the data set deviates from ultrametricity (Meier-Kolthoff et al. 2014). In some cases the preferred value of T has to be selected for maximizing consistency with previous taxonomic work, the foremost example being the 70% (d)DDH threshold to delineate bacterial species (Meier-Kolthoff et al. 2013). But in all other cases it makes sense to choose T so as to minimize the potential of taxonomic inconsistencies related to non-ultrametric data as far as possible. This can be done for bacterial subspecies because by tradition they have not been determined based on a distance or similarity threshold but on a qualitative assessment of few selected phenotypic characters (Staley & Krieg 1984, Wayne et al. 1987). As outlined in the main manuscript, however, it would be advantageous to shift to quantitative, genome-based criteria, for delineating subspecies, too.

Materials

In addition to the *E. coli* data set described in detail in Supplement File S2, we here re-used the data set from Meier-Kolthoff et al. (2014) comprising intergenomic distances calculated with GBDP from genomes of 105 distinct genera of *Archaea* and *Bacteria*. GBDP settings were BLAST+ (Camacho et al. 2009) for obtaining HSPs with a word length of 38 and an e-value cutoff of 10^{-8}, coverage distances (Meier-Kolthoff et al. 2013) and distance formula d_4 (Meier-Kolthoff et al. 2013). These settings were found to be optimal for predicting traditional DDH values from genome sequences (Meier-Kolthoff et al. 2013).

Methods

Partitioning means assigning organisms to one of several non-overlapping groups. In the context of taxonomic classification, this is equivalent to assigning organisms to (formal or informal) taxa of the same rank. Defining a similarity or distance threshold is insufficient for defining a clustering approach if more than two objects are to be clustered (Göker et al. 2009). Non-hierarchical linkage clustering can be varied between single-linkage and complete-linkage clustering. In single-linkage clustering, two clusters are merged if at least one of the distances between the objects in the first cluster is at most as large as the threshold T. This might lead to elongated clusters and the "chaining" effect (Legendre & Legendre 2008). In complete-linkage clustering, two clusters are merged if all of the distances between the objects in the first cluster are at most as large as the threshold T. This leads to spherical, maximally cohesive clusters, but distances to some objects outside such a cluster might be $\leq T$.

The OPTSIL software (Göker et al. 2009) defines, in addition to T, a clustering parameter F that can be varied between 0.0 (non-hierarchical single-linkage clustering) and 1.0 (non-hierarchical complete-linkage clustering). For $F \geq 0.0$, F directly defines the proportion of distances between the objects in two distinct clusters that have to be $\leq T$ for allowing the two clusters to be merged.

To the best of our knowledge, there is no general reason in biology, or even in taxonomic classification, to prefer a certain F value. Like T, F can be optimized by comparisons with a reference partition (Göker et al. 2009). In the case of *E. coli*, the affiliations of strains to the known phylotypes (see main manuscript) may act as such a reference, but their connection to the subspecies rank is unclear. Alternatively, certain statistics can be directly calculated from the clusters, and those parameter combinations be chosen that are optimal according to these statistics. Due to the low number of bacterial subspecies described in the literature (particularly if compared to the number of species), a reference partition cannot directly be used, hence in the following we rely on such statistics to determine optimal parameter combinations.

For each cluster, the cluster isolation (Wirth et al. 1966) is simply the smallest distance of any object within the cluster to an object outside the cluster. The larger, the more separated is the cluster from others and the smaller is the risk of fusion if the sampling of objects is updated or details of the clustering approach are modified. For single-linkage clustering ($F = 0.0$),

by construction cluster isolation must be larger than the distance threshold T that has been applied, but for larger F values cluster isolation can be smaller. Cluster isolation is undefined for a cluster that contains all objects of the clustering.

For each cluster, the cluster cohesion (Wirth et al. 1966) is the proportion of within-cluster distances that is $\leq T$. Cohesion is thus a measure of how spherical a cluster is. So-called cliques have a cluster cohesion of 1.0, as enforced by complete-linkage clustering. Cluster cohesion is undefined for clusters that contain less than three objects, because in all linkage clustering approaches the single distance in two-object clusters must by construction be $\leq T$.

Cluster isolation and cluster cohesion were here calculated as implemented in OPTSIL (Göker et al. 2009). Cluster isolation was only calculated for $F = 1.0$ because this imposes the least constraints on cluster isolation, whereas cluster cohesion was calculated only for $F = 0.0$ because this imposes no constraints on cluster cohesion. That is, those values of T were determined that yielded the highest cluster separation and/or highest cluster cohesion for free, without enforcing them within the chosen clustering approach.

Because cluster isolation and cluster cohesion only measure between-cluster structure or within-cluster structure, respectively, while ignoring the other aspect, and because either metric addresses individual clusters and not entire combinations of distance matrices and inferred clusterings, we devised a further scoring for the purpose of this study. Given a distance matrix, a clustering of the same objects and a distance threshold T, let clustering consistency be defined as the number of within-cluster distances that are $\leq T$ plus the number of between-cluster distances that are $> T$ relative to the total number of entries in the distance matrix. Clustering consistency was calculated from the OPTSIL output using the following R (R Development Core Team 2014) code:

```
# Calculate clustering consistency from 'dists', a squared named distance
# matrix; 'part', a vector or factor whose entries represent the clusters
# and whose names represent the objects; and 'cutoff', a numeric scalar
# which corresponds to the distance threshold used in the clustering that
# yielded this partition.
#
clustering_consistency <- function(dists, part, cutoff) {
  stopifnot(rownames(dists) == colnames(dists),
    setequal(names(part), rownames(dists)))
  ok <- matrix(dists > cutoff, nrow(dists), ncol(dists))
  diag(ok) <- NA
  for (cluster in split(match(names(part), rownames(dists)), part))
    ok[cluster, cluster] <- !ok[cluster, cluster, drop = FALSE]
  mean(ok, na.rm = TRUE)
}
```

Regarding the two extremes of linkage clustering, in the case of single-linkage clustering a consistency < 1.0 can by construction only arise from inter-cluster distances $> T$, whereas

lower than maximum consistency in complete-linkage clustering can by construction only arise from between-cluster distances $\leq T$.

Consistency is relevant regarding the pitfalls emphasized by Meier-Kolthoff et al. (2014) when using pairwise distances or similarities for assessing taxonomic affiliations. For a combination of data set and clustering parameters that yields a clustering consistency of 1.0, such problems do not exist because then by construction a type strain (irrespective of which strain it is) placed within one of the clusters will yield distances $\leq T$ to the other strains placed in this cluster but distances $> T$ to all strains placed in other clusters. That is, linkage clustering on the one hand and pairwise comparison with only the type strain on the other hand will yield the same result in such cases. Only the latter is the taxonomically relevant approach, but only the former is a widespread approach for clustering biological data.

Hence, if the preferred value of T has not to be selected based on the agreement with the traditional taxonomic work – as in the case of the 70% (d)DDH threshold for the delineation of species – it makes sense to choose T so as to maximize clustering consistency (or any other comparable scoring for the usefulness of some clustering result). As mentioned above, this can be done for bacterial subspecies because by tradition they have not been determined based on a distance or similarity threshold but on a qualitative assessment of few selected phenotypic characters.

The dependency on T and F was thus studied for all three measures and for both the *E. coli* and the 105-genera data sets. F was varied between 0.0 and 1.0 with a step width of 0.1; 0.05 was assessed, too, but yielded no differences (data not shown). T was varied between 0.0 and 0.05 with a step width of 0.001. All clusterings were conducted with OPTSIL version 1.5 (available at `http://www.goeker.org/mg/clustering/`). The same settings were also used for clustering optimization with the revised *E. coli* phylotypes as reference partition, maximizing the correspondence between reference and clustering partition as measured using the modified Rand index (Göker et al. 2009).

We also had a look at the frequency distribution of dDDH values in the data sets. As our conclusions are not directly based on them, they are presented in the appendix only.

Results

Results from clustering optimization are shown in Table S3-1. The highest modified Rand index (MRI), 0.9752, was obtained for $F = 0.9$ and $T = 0.0135$. These settings yielded 32 clusters, way more than the number of phylotypes in the reference partition, which comprised 21 groups, i.e. 15 *E. coli* and six outgroup groups.

Expectedly, cluster isolation showed no relevant dependency on F (data not shown), hence we abstained from depicting it. Conversely, cluster cohesion was trivially dependent on F, with monotonically increasing average values until achieving maximum cohesion throughout for $F = 1.0$ (data not shown).

F	Highest MRI	Best T	# clusters
0	0.9626	0.0099	36
0.05	0.9723	0.0107	35
0.1	0.9695	0.011	36
0.15	0.9626	0.0112	36
0.2	0.9626	0.0113	36
0.25	0.9626	0.0114	36
0.3	0.9627	0.0117	35
0.35	0.9627	0.0118	35
0.4	0.9627	0.0119	35
0.45	0.9627	0.0119	35
0.5	0.9627	0.0119	35
0.55	0.9627	0.012	35
0.6	0.9627	0.012	35
0.65	0.9626	0.0125	34
0.7	0.9626	0.0126	34
0.75	0.9626	0.0127	34
0.8	0.9626	0.0127	34
0.85	0.9626	0.0129	34
0.9	0.9752	0.0135	32
0.95	0.9656	0.0136	33
1	0.9425	0.0183	19

Table S3 - 1: Highest modified Rand index (MRI) values in dependency of F when using the revised phylotypes as reference partition. The best T and the resulting number of clusters for each F values are also indicated.

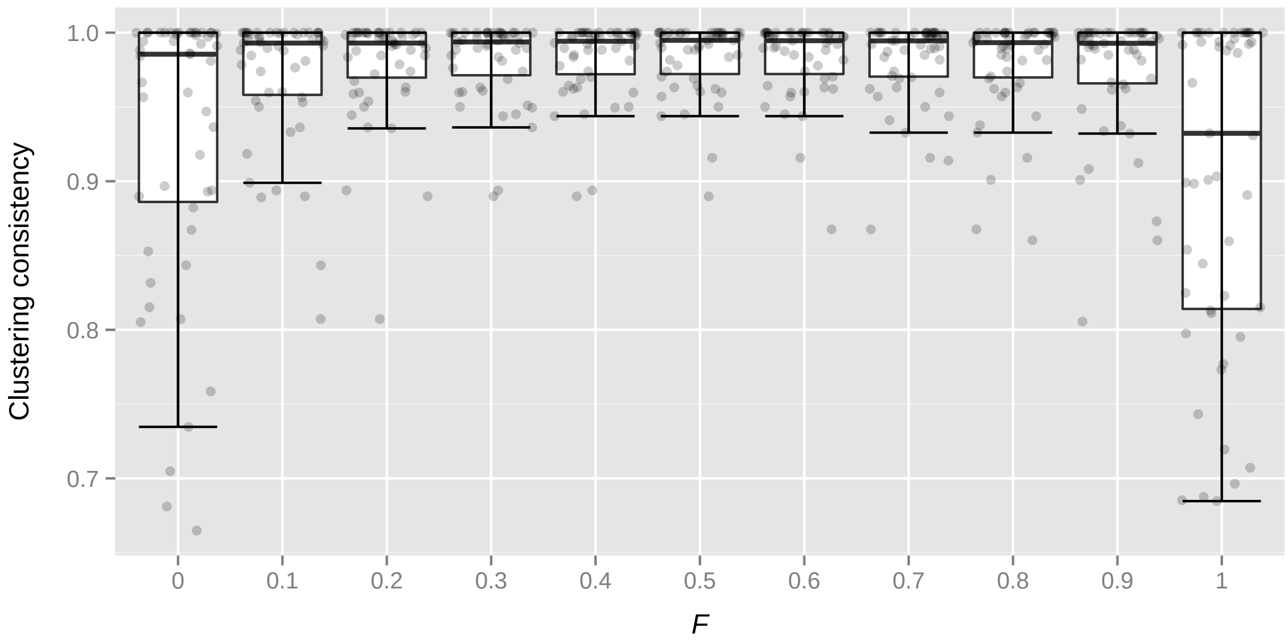

Figure S3 - 1: Box plots depicting the dependency of the clustering consistency on F for the *E. coli* data set. Note the slightly different scale on the y-axis compared to Figure S3-2.

The dependency of the clustering consistency on F is depicted in Figure S3-1 for the *E. coli* data set. Apparently maximum consistency is obtained for the F values around 0.5, with average consistency decreasing for lower as well as higher F values.

The 105-genera data set, depicted in Figure S3-2, yielded the same general tendency. In contrast to the *E. coli* data set, however, apparently much more parameter combinations obtained maximum consistency, thus yielding strongly skewed overall distributions. Nevertheless, the lowest observed consistencies were lower as in the case of the *E. coli* data set.

Figure S3-3 shows the dependency of the average cluster isolation on the distance threshold T for the *E. coli* data set. Expectedly, cluster isolation is monotonically decreasing with decreasing T. Sudden drops hinting at cluster instability, however, occur at approximately 75% and at approximately 85% dDDH. In the area around 75% dDDH a strong sensitivity to F is also observed.

For the 105-genera data set, the dependency of cluster isolation on T is depicted in Figure S3-4. Here, neither sudden drops nor strong sensitivity to F are apparent.

Figure S3-5 depicts the dependency of the average cluster cohesion on the distance threshold T for the *E. coli* data set. Obviously, cluster cohesion is low only for low F values and trivially maximum for $F = 1.0$. A more interesting observation, however, is that for low F values cluster cohesion strongly decreases with T approaching 0.0, with additional local minima slightly below 90% dDDH, at between 90% and 95% dDDH, and at about 85% dDDH and slightly higher. Conversely, local maxima are apparent at about and slightly above 80% dDDH, slightly below 90% dDDH, and at about 95% dDDH. At 70% dDDH (the species boundary), cluster cohesion

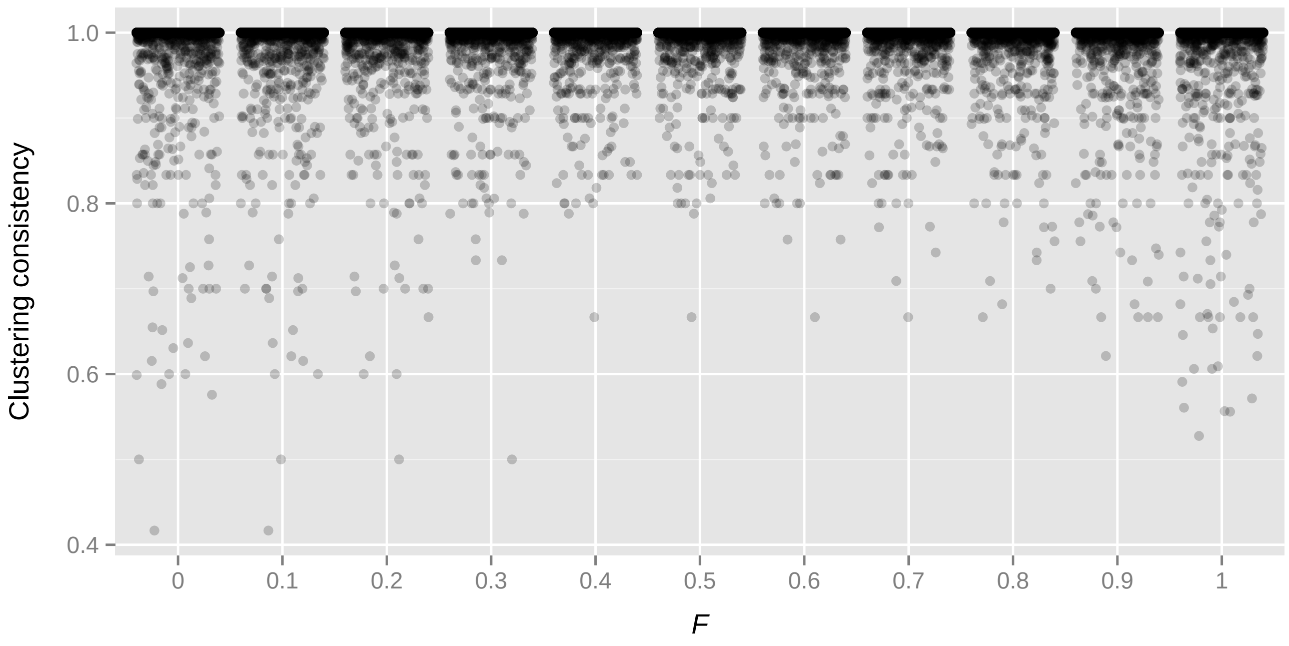

Figure S3 - 2: Box plots depicting the dependency of the clustering consistency on F for the 105-genera data set. Note the slightly different scale on the y-axis compared to Figure S3-1.

is maximum for all F values, but this might just be an effect of strain sampling because the focus was on a single species, *E. coli*.

The cluster-cohesion results for the 105-genera data set are shown in Figure S3-6. Trimmed means had to be used for visualization because most cohesions were maximum. In agreement with the *E. coli* data set, a local minimum is observed at about 85% dDDH, followed by a set of local maxima around 80% dDDH, and a global decrease for T approaching 0.0 interrupted by further local maxima at about 92% dDDH and slightly above 95% dDDH. Interestingly, clustering cohesion is almost maximum throughout at 70% dDDH but somewhat higher slightly below, more strikingly lower slightly above 70% dDDH.

The dependency of the clustering consistency on T is shown in Figure S3-7 for the *E. coli* data set. Consistency is partially very low for the extreme F values 0.0 and 1.0 (see also Figure S3-1). For the other F values, local maxima are observed at 70% dDDH and higher, slightly below 80% dDDH, slightly below 85% dDDH, slightly below 90% dDDH, and for T approaching 0.0.

In contrast to the *E. coli* data set, the 105-genera data sets yielded consistency values of 99.9% or higher throughout, as depicted in Figure S3-8. Despite this overall way more optimistic picture, some analogies to the *E. coli* data can be seen. These include the relatively low performance of extreme F values (as more clearly visible in Figure S3-2) and the local maximum at slightly below 80% dDDH.

To address the question why the 105-genera data set shows on average much higher consistency values than the *E. coli* data set, we examine its dependency on the sampling of genomes per

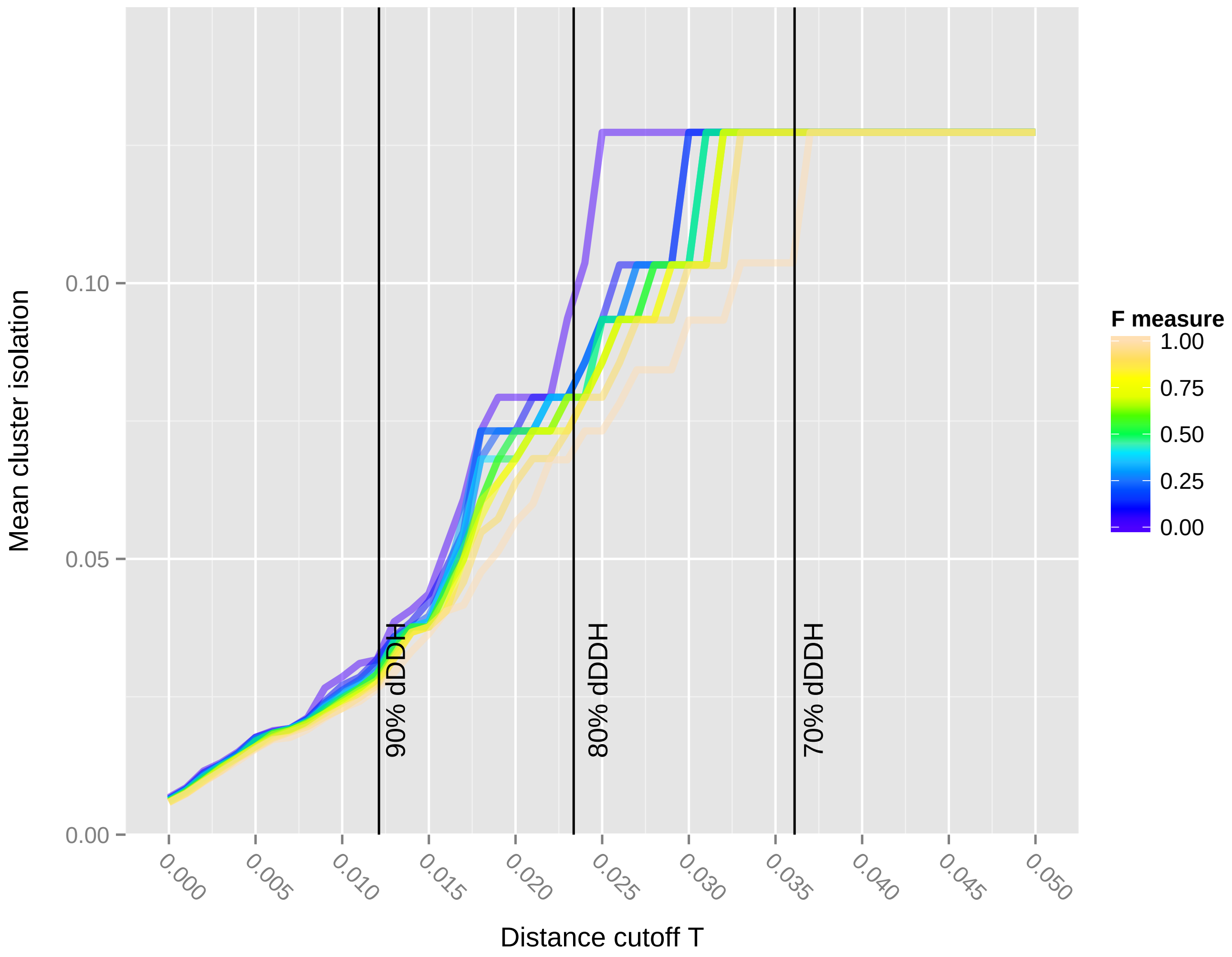

Figure S3 - 3: Dependency of the cluster isolation (averaged over each clustering) on the distance threshold T for the *E. coli* data set. Colors indicate the distinct F values used. Note the slightly different scale on the y-axis compared to Figure S3-4.

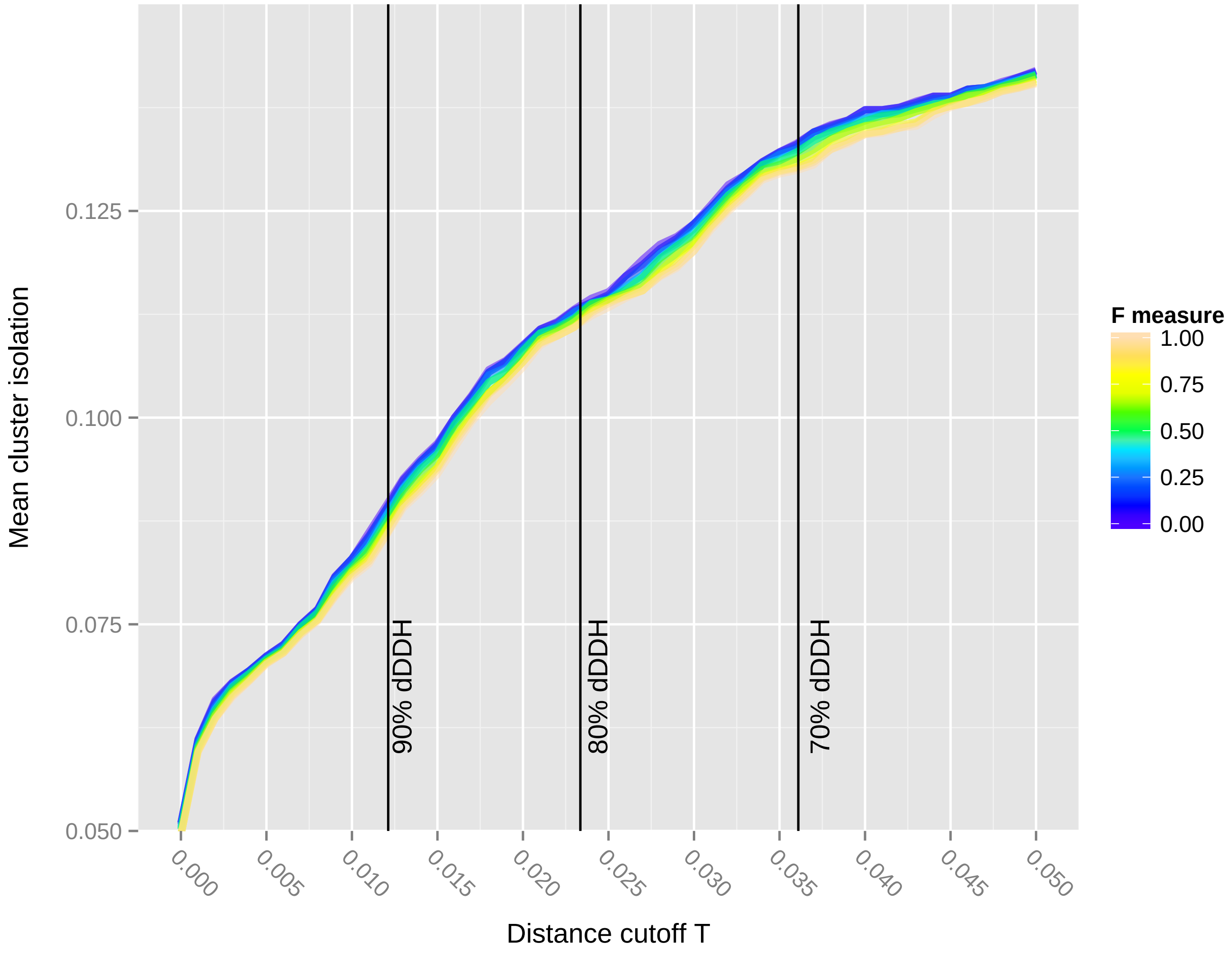

Figure S3 - 4: Dependency of the cluster isolation (averaged over each clustering and all data sets) on the distance threshold T for the 105-genera data set. Colors indicate the distinct F values used. Note the slightly different scale on the y-axis compared to Figure S3-3.

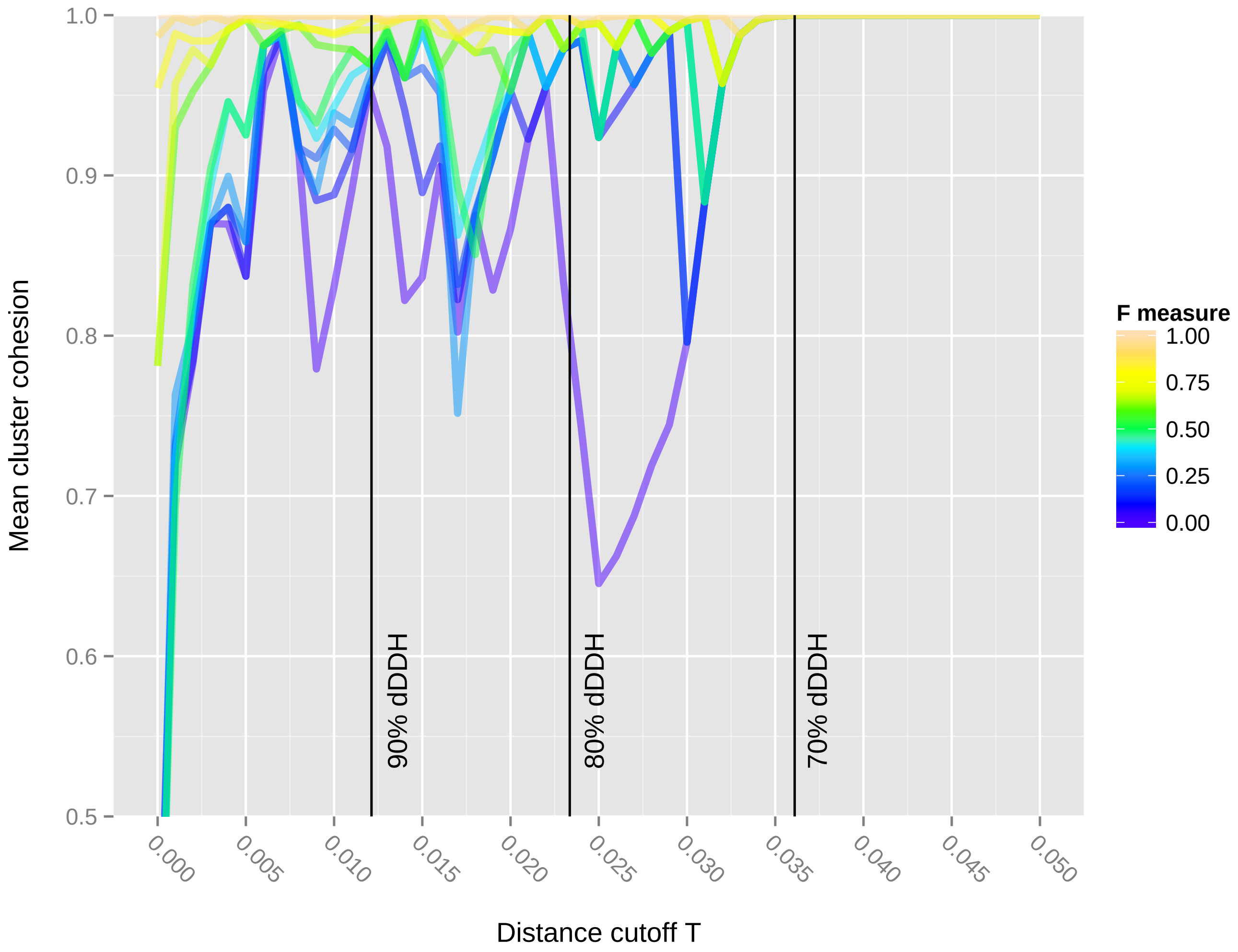

Figure S3 - 5: Dependency of the cluster cohesion (averaged over each clustering) on the distance threshold T for the *E. coli* data set. Colors indicate the distinct F values used. Note the strongly different scale on the y-axis compared to Figure S3-6.

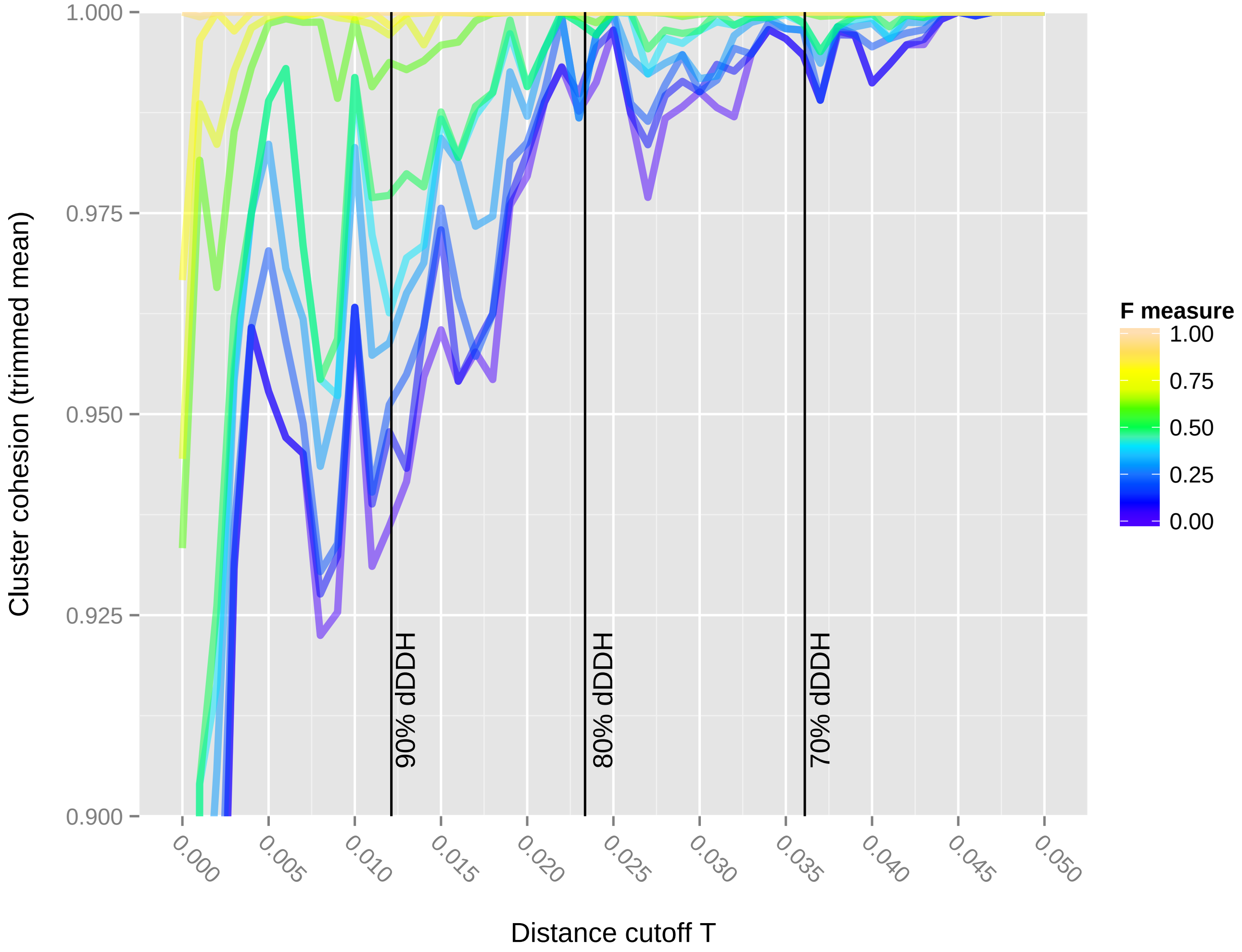

Figure S3 - 6: Dependency of the cluster cohesion (averaged over each clustering and all data sets) on the distance threshold T for the 105-genera data set. Colors indicate the distinct F values used. Note the strongly different scale on the y-axis compared to Figure S3-5. In contrast to Figure S3-5, 0.05-trimmed means were used.

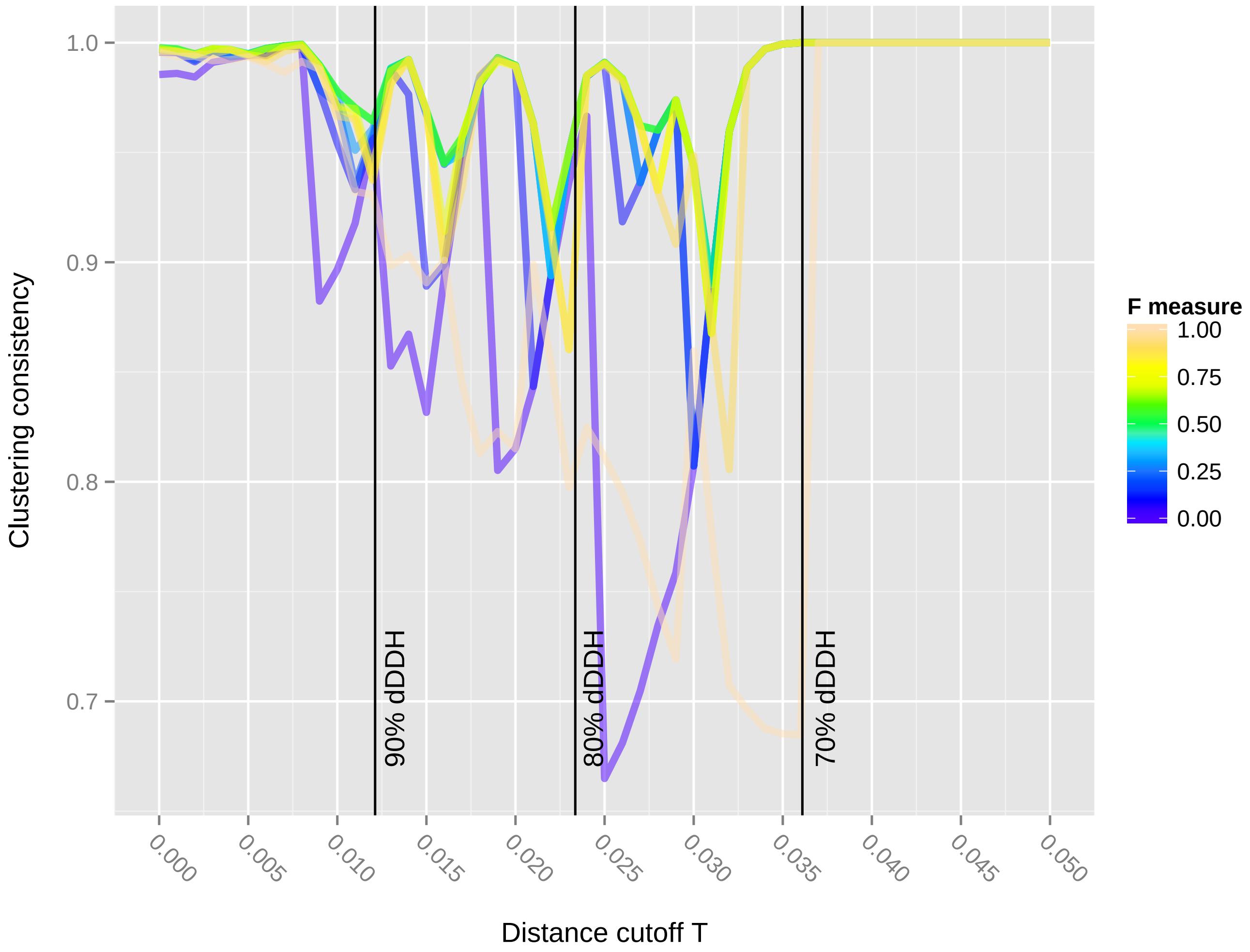

Figure S3 - 7: Dependency of the clustering consistency on the distance threshold T for the *E. coli* data set. Colors indicate the distinct F values used. Note the strongly different scale on the y-axis compared to Figure S3-8.

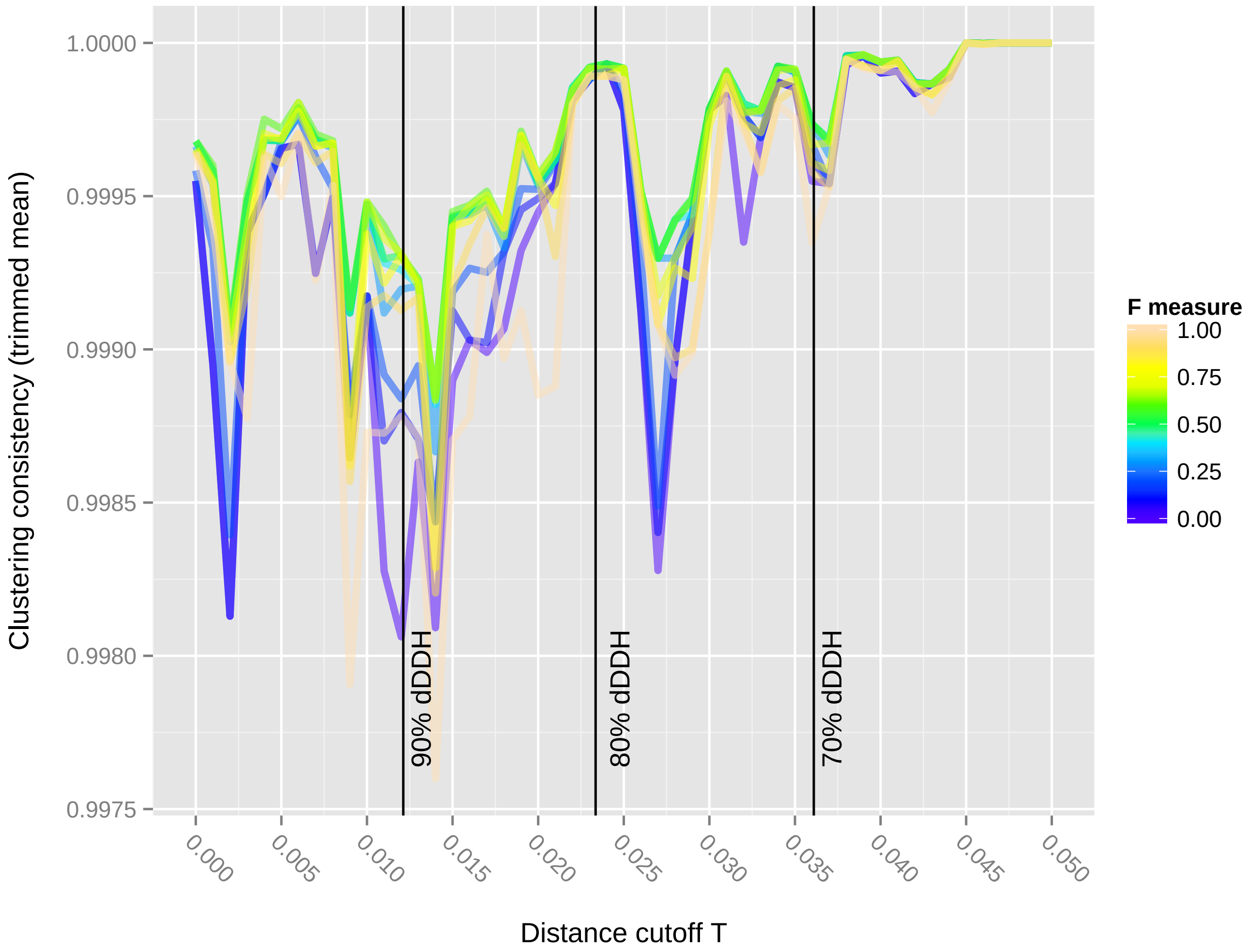

Figure S3 - 8: Dependency of the cluster consistency (averaged over all data sets) on the distance threshold T for the 105-genera data set. Colors indicate the distinct F values used. Note the strongly different scale on the y-axis compared to Figure S3-7. In contrast to Figure S3-7, 0.05-trimmed means were used.

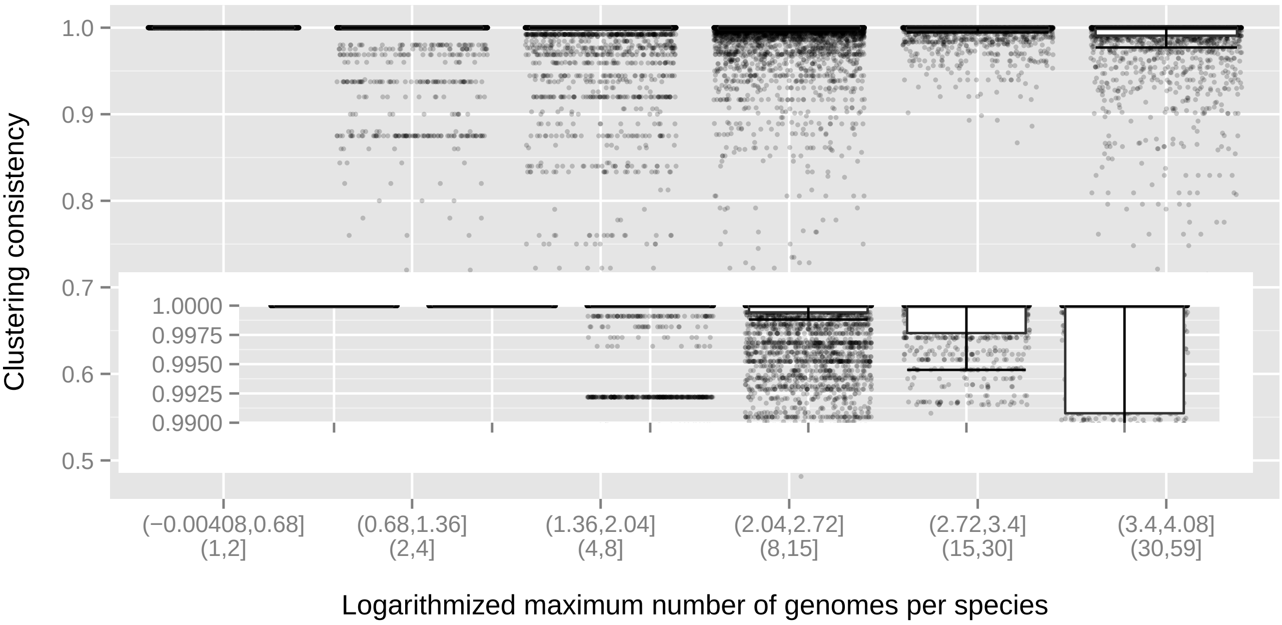

Figure S3 - 9: Dependency of the cluster consistency (averaged over all distance thresholds T and all F values used) set on the maximum number of genomes per species in each genus within the 105-genera data set. The maximum number of genomes per species was categorized after logarithmic conversion. The x-axis annotation shows the logarithmised values in the upper row, the original values in the lower row.

species, as shown in Figure S3-9. Data sets with more genomes per species indeed show lower median consistency values, in accordance with the observation for the *E. coli* data set (which contains more genomes per species than the largest subset of the 105-genera data set).

Given the decrease of consistency relative to the data set size (as measured by the maximum number of genomes per species) we re-assessed the dependency of the consistency on T and all F using weighted means. As weights, we used the logarithmised maximum number of genomes per species, thus emphasizing the larger subsets (better sampled genera) in the 105-genera data set. The result is shown in Figure S3-10. The apparent drop in average consistency compared to Figure S3-8 is as expected due to the weighting. The peak at around 79% dDDH remains the most prominent one in this area. Higher peaks below 70% dDDH do occur now, but only at about 93% dDDH. The location of peaks and depressions is quite similar to the one depicted in Figure S3-7 for *E. colit* with the exception of an additional decrease around 92% dDDH.

An analysis of the frequency distribution of the dDDH values is provided in the appendix. It is not in conflict with data presented above but we do not directly base any conclusions on it; see the appendix for details.

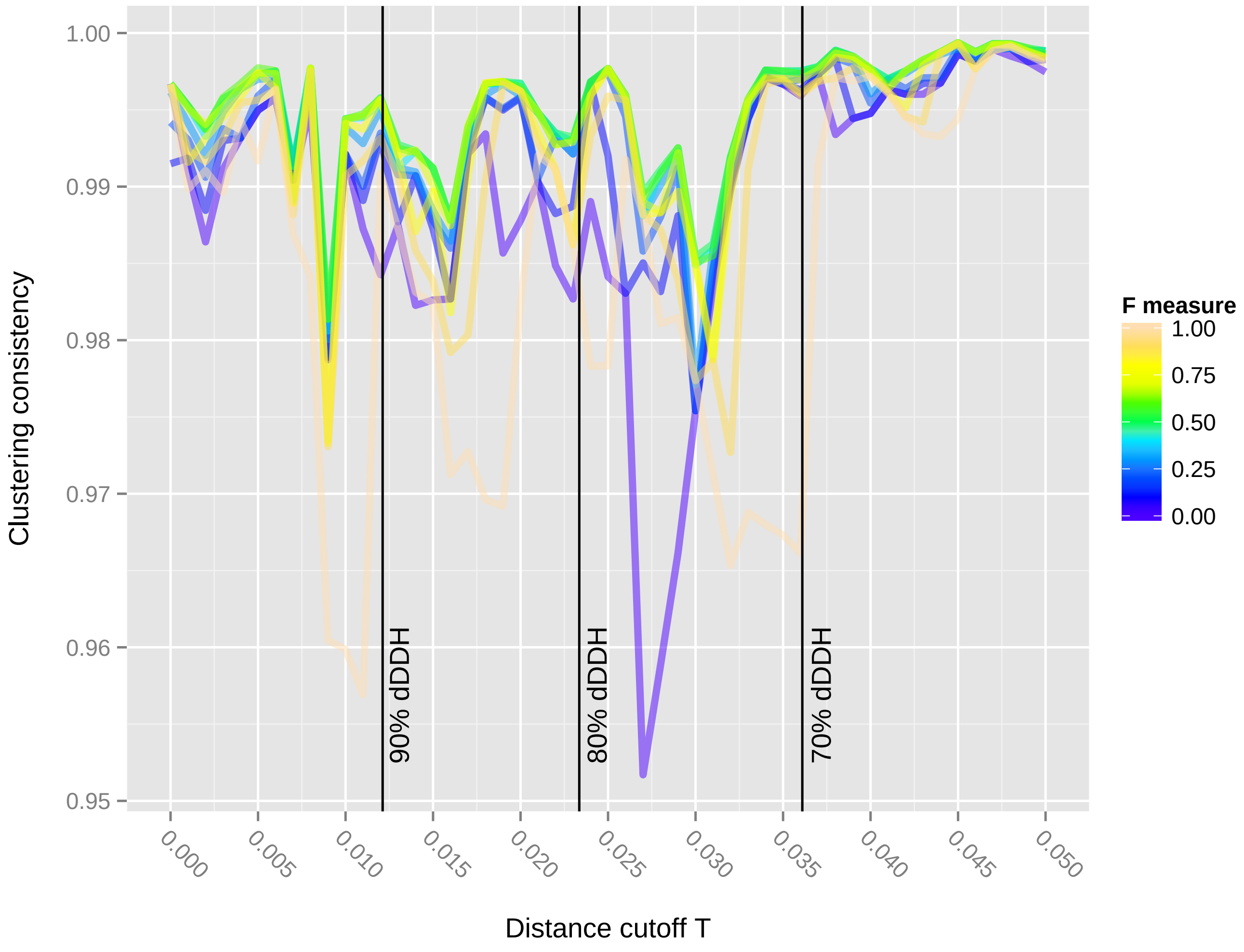

Figure S3 - 10: Dependency of the cluster consistency (averaged over all data sets) on the distance threshold T for the 105-genera data set. Colors indicate the distinct F values used. Note the different scale on the y-axis compared to Figure S3-8. In contrast to Figure S3-8, weighted means were used.

Discussion

The clustering optimization approach, much like the cluster statistics for the phylotypes listed in the main manuscript, indicated that even the revised *E. coli* phylotypes are only partially homogeneous regarding their between- and within-cluster intergenomic distances, if calculated with nucleotide GBDP.

For the other purpose of this study, i.e. to determine optimal clustering settings for subspecific bacterial lineages, the overall trends appeared to be more clearly visible using the clustering consistency measure than either cluster isolation or cluster cohesion. The analysis of the latter, however, did not yield strong discrepancies to using clustering consistency, a possible exception being the strong decrease of cluster cohesion towards $T = 0.0$. This might simply be caused by the impossibility to calculate cohesion for clusters containing less than three objects, which of course get more frequent if the clustering threshold is decreased. If so, avoiding artifacts like that might be an advantage of the clustering consistency metric.

Regarding clustering consistency, as striking outcome of the analysis of the 105-genera data set is that consistency was close to maximum throughout. That is, for most combinations of data set and clustering parameters, modifying the parameters did not significantly lower the consistency, in accordance with the results of Meier-Kolthoff et al. (2014) who analyzed triplets of organisms at the 70% dDDH boundary only. This is good news but of little help for selecting an optimal threshold T for delineating subspecies. Yet given the shape of the curves in Figure S3-8, either none or only a single recommendation can be made, i.e. to apply a boundary of 79-80% dDDH. Other values of T either yielded lower average consistencies or were to close to, or above, 70% dDDH, which is already reserved for the species rank.

Moreover, our results indicate that clustering consistency is effected by sampling. The higher the number genomes per species, the larger the differences between distinct T and F values regarding the consistency of the clustering. This result is not unexpected, as a lower number of genomes is likely to cause a lack of variety within the distance matrices. This in turn causes a larger variety of T and F values to yield the same clustering, and with fewer discrepancies, as the distances that might lower the consistency are less likely to be present in the matrix. It follows that the results for *E. coli* are actually in line with those of the 105-genera data set despite the differences in average consistency, as the *E. coli* data set mainly differs regarding its sampling size. It also follows that the choice of clustering parameters matters, as increased sampling increases the need for parameters that still yield a high clustering consistency.

Indeed, results obtained with the *E. coli* data set, which showed much greater variety regarding clustering consistency in dependence of T, agree with the suggestion based on the 105-genera data set also regarding optimal thresholds. The *E. coli* data showed a local maximum at about 79% dDDH (Figure S3-7). Other local maxima where present, as well as an even stronger increase towards 100% dDDH, but these were not observed within the 105-genera data set. A number of other arguments also favor a boundary around 79-80% dDDH:

- Lower boundaries would be too close to 70% dDDH and thus for most known species yield either no distinct subspecies at all or only hardly distinguishable ones.

- Given the currently rare description of subspecies, boundaries higher than 80% dDDH might inflate the number of described subspecies.

- Higher boundaries than 80% dDDH, such as 84% (Figures S3-7, S3-8, S3-10) or 93% (Figures S3-7, S3-10) might later on be reserved for varieties or other ranks lower than subspecies rank.

- Values approaching 100% dDDH might just represent clones, or even genome sequences obtained several times from the same strain.

Conclusions

In order to foster the transition to a quantitative, genome-based delineation of subspecies, we recommend a dDDH boundary of 70-80%. In addition to other criteria, this threshold yields the highest clustering consistency and thus the least amount of those problems that are generally related to all uses of pairwise distances or similarities for taxonomic purposes (Meier-Kolthoff et al. 2014).

References

Camacho, C., Coulouris, G., Avagyan, V., Ma, N., Papadopoulos, J., Bealer, K., Madden, T. BLAST+: architecture and applications. BMC Bioinformatics 10: 421, 2009 (doi:10.1186/1471-2105-10-421).

Göker, M., García-Blázquez, G., Voglmayr, H., Tellería, M.T., Martín, M.P. Molecular taxonomy of phytopathogenic fungi: a case study in *Peronospora*. PLoS ONE 4: e6319, 2009 (doi:10.1371/journal.pone.0006319).

Legendre, P., Legendre, L. Numerical ecology, 2nd English edition. Amsterdam: Elsevier Science BV, 2008. pp xv+853.

Meier-Kolthoff, J.P., Auch, A.F., Klenk, H.-P., Göker, M. Genome sequence-based species delimitation with confidence intervals and improved distance functions. BMC Bioinformatics 14: 60, 2013 (doi:10.1186/1471-2105-14-60).

Meier-Kolthoff, J.P., Klenk, H.-P., Göker, M. Taxonomic use of the G+C content and DNA:DNA hybridization in the genomic age. International Journal of Systematic and Evolutionary Microbiology 64: 352-356, 2014 (doi:10.1099/ijs.0.056994-0).

R Development Core Team. R: a language and environment for statistical computing. R Foundation for Statistical Computing, Vienna, Austria, 2014 (http://r-project.org/).

Staley, J., Krieg, N.R. Bacterial classification I. Classification of procaryotic organisms: an overview. In: Krieg, N.R., Holt. J.G. (editors), Bergey's Manual of Systematic Bacteriology, vol. 1, The Williams & Wilkins Co, Baltimore, 1984, p. 1-4.

Wayne, L.G,, Brenner, D.J., Colwell, R.R., et al. Report of the ad hoc committee on reconciliation of approaches to bacterial systematics. International Journal of Systematic Bacteriology 37:463-464, 1987 (doi:10.1099/00207713-37-4-463).

Wirth, M., Estabrook, G.F., Rogers, D.J. A graph theory model for systematic biology, with an example for the *Oncidiinae* (*Orchidaceae*). Systematic Zoology 15: 59-69, 1966.

Appendix: Frequency distribution of dDDH values

It is tempting to select distance or similarity thresholds from the gaps or at least depressions in histograms of empirically observed distances or similarities. If the data are metric, such gaps might indeed indicate "real" boundaries in the data.

We abstained from basing our main conclusions on such frequency distributions, however, because the distance or similarity threshold itself tells nothing about the additional clustering parameters to be used such as F. If a certain clustering approach is envisaged, it is more informative to conduct the clustering under a given distance or similarity threshold and assess the result. For instance, gaps or depressions in histograms do not address the concerns raised by Meier-Kolthoff et al. (2014). Thus it is preferable to directly measure clustering consistency to tackle those issues.

Whereas they might not be relevant for just the search for gaps in the distribution of distances or similarities, some other features of their matrices should be kept in mind, too. Distance and similarity matrices are highly redundant, as the number of values they contain is proportional to the squared of the number of objects (which are genomes in our case). In contrast, the number of branches in a phylogenetic tree was only proportional to the number of objects. The single entries in distance and similarity matrices are not statistically independent from each other, as they are constrained by (near-)metricity or even (near-)ultrametricity (Meier-Kolthoff et al. 2014). Thus given the distances d_{AB} and d_{AC} between objects A, B and C, the distance d_{BC} cannot freely vary. Thus distance or similarity matrices cannot directly be analyzed with the usual statistical means. For these reasons, one might expect frequency distributions of similarities or distances to be strongly affected by sampling.

Having said this, we hasten to add that the frequency distributions depicted in Figures S3-11 and S3-12 are not at all in conflict with the analysis of clustering consistencies of the same data. The gaps or depressions are at the expected positions, and readers who prefer analyzing such frequency distributions might well interpret this as evidence that the suggested clustering threshold represents something "real" in the data.

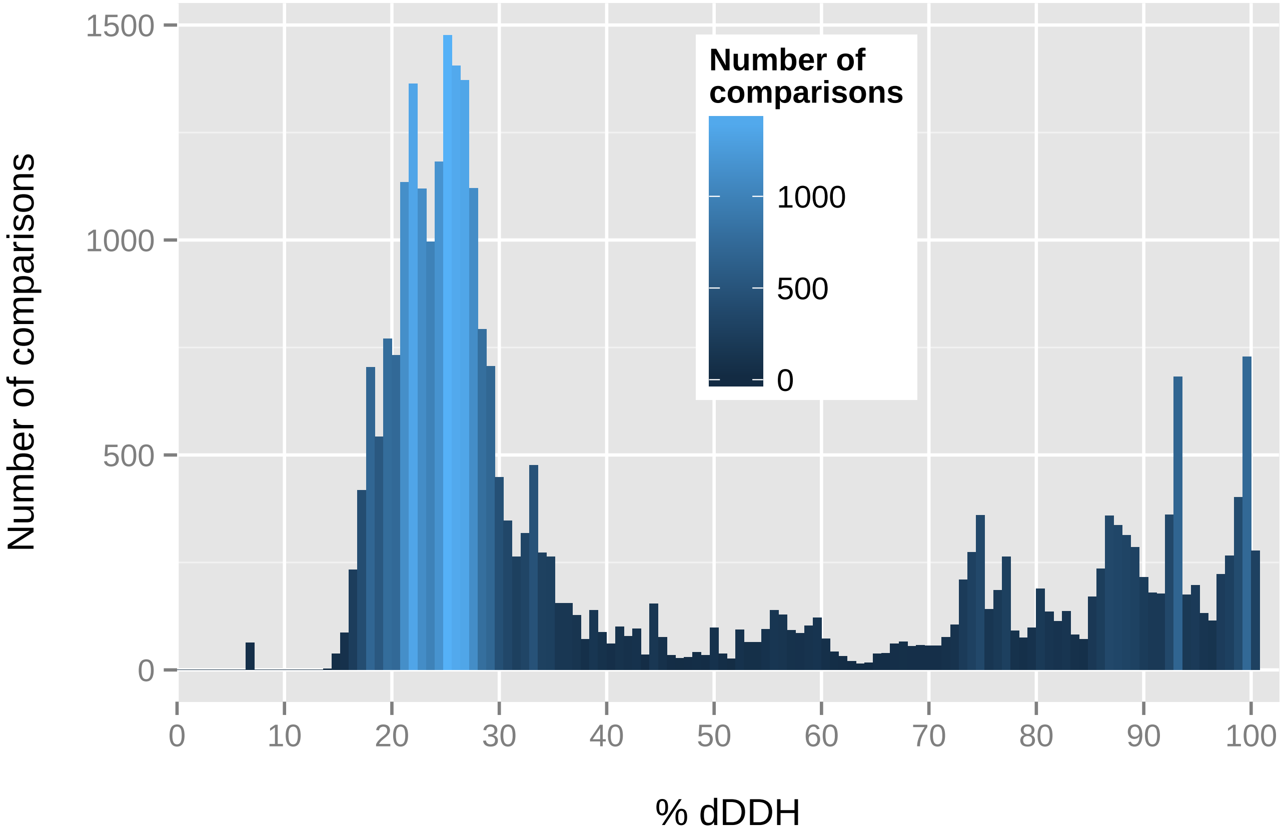

Figure S3 - 11: Frequency distribution of dDDH values in the 105-genera data set.

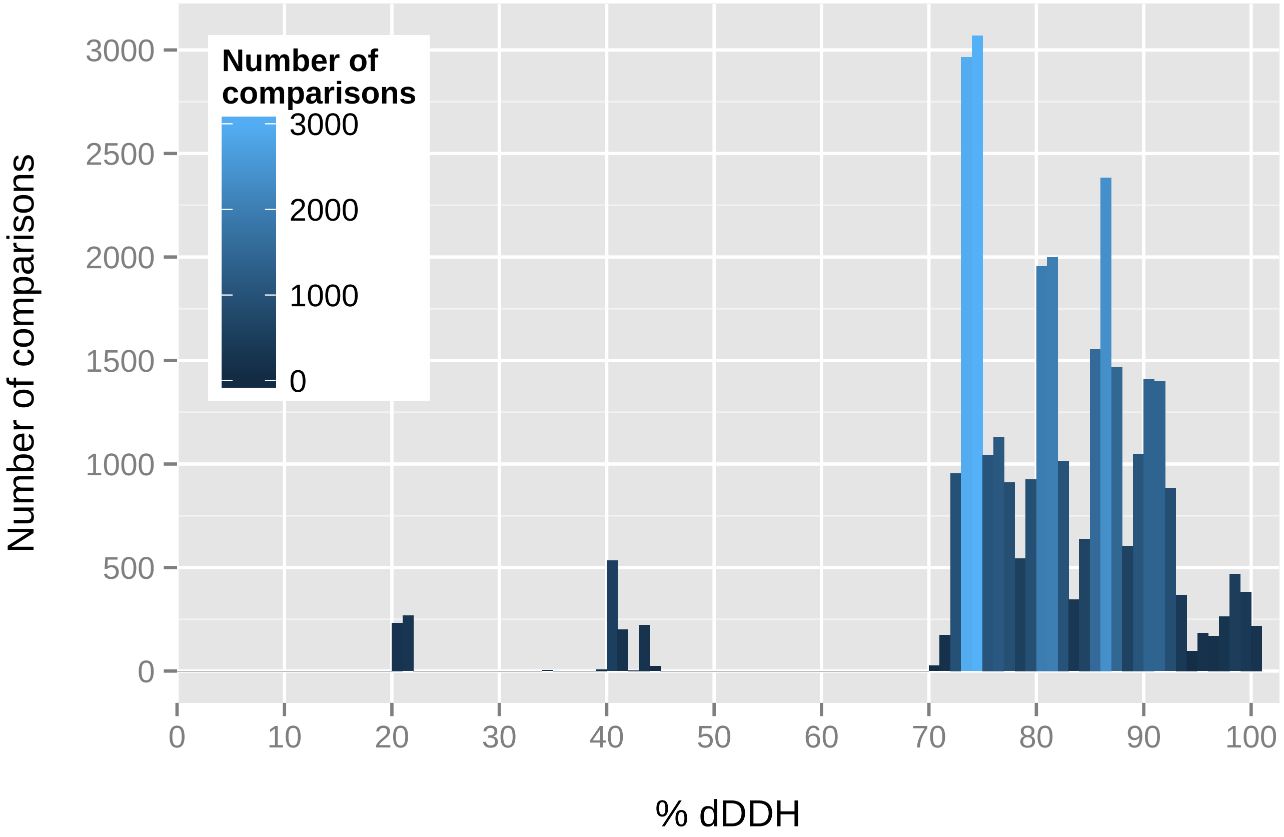

Figure S3 - 12: Frequency distribution of dDDH values in the *E. coli* data set. The same picture is included in supplementary file S1.
